# Initial Body Weight as an Important Factor for Improving the Reliability and Translational Relevance of the Preclinical Monocrotaline-Induced Rat Pulmonary Hypertension Model

**DOI:** 10.3390/ijms26188916

**Published:** 2025-09-12

**Authors:** Patryk Remiszewski, Piotr Ryszkiewicz, Marta Baranowska-Kuczko, Anna Pędzińska-Betiuk, Krzysztof Mińczuk, Monika Kloza, Jolanta Weresa, Tomasz Hutsch, Barbara Malinowska

**Affiliations:** 1 Department of Experimental Physiology and Pathophysiology, Medical University of Bialystok, 15-222 Bialystok, Poland; patryk.remiszewski@umb.edu.pl (P.R.); piotr.ryszkiewicz@umb.edu.pl (P.R.); marta.baranowska-kuczko@umb.edu.pl (M.B.-K.); anna.pedzinska-betiuk@umb.edu.pl (A.P.-B.); krzysztof.minczuk@umb.edu.pl (K.M.); monika.kloza@umb.edu.pl (M.K.); jolanta.weresa@umb.edu.pl (J.W.); 2 Department of Pathology and Veterinary Diagnostics, Institute of Veterinary Medicine, University of Life Sciences, 02-776 Warsaw, Poland; tomasz_hutsch@sggw.edu.pl; 3 Veterinary Diagnostic Laboratory ALAB Bioscience, 00-739 Warsaw, Poland

**Keywords:** ambrisentan, initial body weight, methodological rigor, monocrotaline, pulmonary hypertension, rat, tadalafil

## Abstract

Animal preclinical experiments in pulmonary hypertension (PH) need to be conducted with detailed methodological rigor to improve their translational relevance. One of its crucial yet insufficiently studied aspects is animal body weight (BW). Thus, our study aimed to examine the influence of initial BW on the severity of PH development induced by monocrotaline (MCT) and the effectiveness of the reference combined therapy (ambrisentan and tadalafil given for 21 days). Male rats were divided into three weight Sets: Set I (200–219 g); Set II (220–239 g); and Set III (240–259 g), after which, MCT-PH was induced. The measurements taken included in vivo echocardiographic evaluations, ex vivo functional experiments (on isolated right ventricle papillary muscles and pulmonary arteries), and histological and morphometric assessments. In all three Sets of animals, we noticed evidence of PH development. More pronounced changes confirming the severity of PH were observed in Set II compared to Sets I and III. The effectiveness of the reference therapy was also most evident in Set II, where the reversal of PH-related aggravations was best documented. We demonstrated that both the severity of MCT-induced PH in rats and the effectiveness of the reference combined therapy strongly depend on the animals’ initial BW.

## 1. Introduction

Pulmonary arterial hypertension (PAH) is a severe, multi-factorial cardiopulmonary disease progressing with changes in the pulmonary circulation (remodeling, constriction, and endothelial dysfunction) causing the development of right ventricular (RV) hypertrophy and RV failure that eventually leads to death [1,2]. Although mortality rates have decreased over the last two decades, the 5-year survival rate is still not satisfactory [3]. Despite intensive research on the disease’s pathomechanisms and pharmacotherapy, PAH is still considered incurable. The currently approved drugs for PAH are primarily targeted towards the reduction in pulmonary vasomotor tone [4]. For instance, the combination of endothelin 1 (ET-1) antagonist ambrisentan and phosphodiesterase 5 (PDE-5) inhibitor tadalafil is considered the gold standard for most patients with PAH [5]. The treatment of RV failure, which occurs in PAH, lacks effective therapeutic strategies, as drugs for left heart failure are not quite beneficial [6]. Thus, searching for new therapeutic approaches is of key importance [7].

Animal experiments, as a part of preclinical studies, are a crucial step in the investigation of molecular mechanisms underlying the disease and identification of new actionable therapeutics. Several small animal models are frequently used in the study of PAH [8,9,10,11,12,13,14,15,16,17]. Unfortunately, promising results for many drugs during preclinical evaluation had only low success during subsequent clinical testing. Such a translational gap is often called ‘the Valley of Death’ [8,18]. Therefore, the search for new effective therapies against PAH and RV failure should comprise not only the discovery of new molecular targets for innovative and successful therapies but should also precisely define the criteria for improving the preclinical assessment of beneficial therapy. The detailed standards and methodological rigor in PAH preclinical and translational research have been described by Provencher et al. [19], who underlined the importance of rigorous study designs, methodological standardization, appropriate data interpretation, adopting statistical analysis plans, and the transparent reporting of preclinical confirmatory studies.

Pulmonary hypertension (PH) induced by the administration of monocrotaline (MCT) is the oldest and one of two (in addition to chronic hypoxia exposure) so-called classical experimental models of this disease [8,9,10,11,12,13,14,15,16,17]. It has been widely used for nearly 60 years due to its technical simplicity, reproducibility, and relatively low cost. Based on the above and the number of research papers (over 2000 in PubMed over the last 20 years), investigators often favor the MCT model. MCT-induced PH has been beneficial both as an in vivo model of severe PAH, allowing for the elucidation of pathobiological mechanisms, as well as also being used extensively as a preclinical model of PAH to test potential therapeutic agents.

The severity of experimental PH, as well as the therapeutic potency of investigated drugs, depends on many aspects, such as the experimental model, species (rats vs. mice), strain (Wistar, Sprague Dawley, or Fisher rats), sex, animal age, anesthetic(s) used during the final outcome assessment, the length of the period dedicated to the development of PH, and the study protocol (preventive or therapeutic) [8,10,11,12,13,16,17,20,21,22]. The above mentioned factors are also valid for the MCT-induced PH [11,23,24,25]. However, one issue—the influence of initial body weight (BW), mentioned by Sztuka and Jasińska-Stroschein [13] in their systematic review and meta-analysis based on data from 6126 animals, has not been examined in detail. Importantly, in our previous papers, we observed that a few-week difference in the age of rats may result in different outcomes, both in systemic [26] and pulmonary hypertension [27,28]. Thus, we noticed that the same dose of MCT resulted in mild PH [28] in younger animals and only early-stage PH [27] in only 3–4 weeks older Wistar rats. Similarly, Krstic et al. [25], in their review, stated that the BW of the rats at the time of MCT injection, rather than the age per se, is the key to the rate of RV disease progression since rats injected at ~350 g BW did not develop heart failure until 5–6 weeks post-injection, whereas rats injected at ~200 g BW transitioned to RV failure at around 3–4 weeks post-injection. The above study suggests that a lower initial BW determines more severe PH.

Our central hypothesis is that the initial BW plays a significant role in influencing both disease progression and treatment response in male rats. This could have important implications for subject stratification and the personalization of treatment approaches in preclinical models of PH. Taking the above under consideration, the aim of our study was to examine the influence of initial BW on (1) the severity of PH development induced by MCT injection in rats and (2) the effectiveness of the reference combined therapy with ambrisentan and tadalafil.

## 2. Results

### 2.1. General

As shown in Table 1, animals were divided into three Sets based on their initial BW (on day 0): 200−219 g (Set I), 220−239 g (Set II), and 240−259 g (Set III). Control (CTR + veh), pulmonary hypertensive (PH) (MCT + veh), and treated (MCT + AMB + TAD) animals did not differ in BW within each of these Sets. Nevertheless, the initial BW of rats was higher in Set II than in Set I, and the highest in Set III.

During the 29 days of experiments, animals from all groups gained BW (Table 1). The strongest and most continuous growth was observed in the CTR + veh group (day 29 vs. day 0; by about 55, 40, and 36% in Set I non-significantly and significantly in Sets II and III, respectively). A gradual decrease in BW gain, and, eventually, a reduction in total BW (vs CTR + veh on day 29) was observed in MCT-induced PH rats (in which BW between day 0 and day 29 increased by 33, 23, and 17% in Sets I, II, and III, respectively). Thus, increases in BW obtained on day 29 compared to day 0 (BW_29_−BW_0_) in MCT-treated groups were reduced by 40, 42, and 51% compared to respective control groups in Set I, II, and III, respectively. AMB + TAD treatment reversed such a fall in BW only in Set II. When considering a bone/skeletal growth indicator, tibia length, slight increases (by about 3%) on day 29 were detected only in MCT + AMB + TAD groups (Set II vs. Set I and Set III vs. Set I).

### 2.2. Influence of PH and Drug Therapy on RVSP, mPAP, Fulton’s Index, Blood Oxygen Saturation, and Survival Rates

To assess the influence of initial BW on the most crucial indicators of the severity of PH, we primarily measured RV systolic pressure (RVSP), mean pulmonary artery pressure (mPAP), Fulton’s index, blood oxygen saturation, and survival rates.

**Figure 1:**
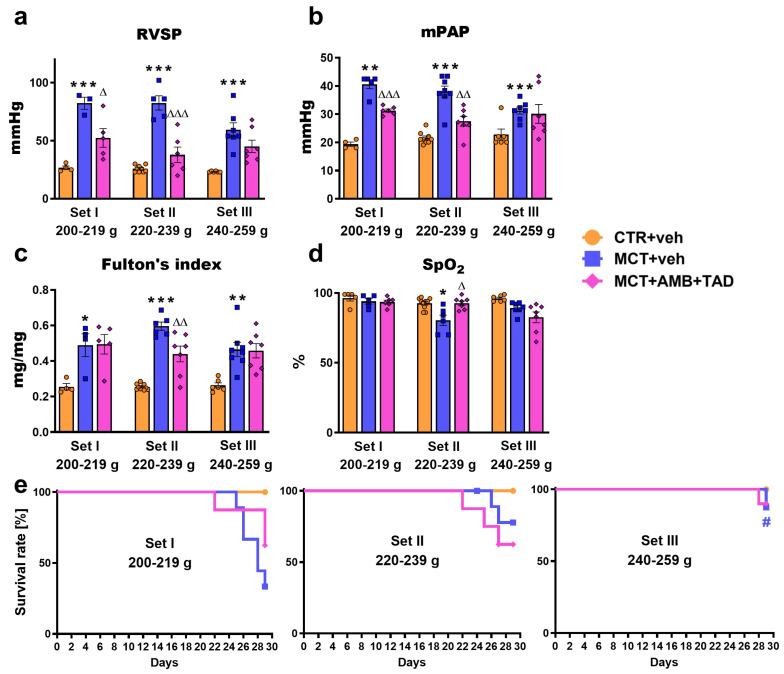
Influence of pulmonary hypertension (PH) and treatment with a combination of ambrisentan (AMB) and tadalafil (TAD) or their vehicle (veh) on right ventricular systolic pressure (RVSP; (**a**)), mean pulmonary artery pressure (mPAP; (**b**)), Fulton’s index (**c**), blood oxygen saturation (SpO_2_; (**d**)), and survival rate (**e**) of monocrotaline (MCT)-induced PH rats and their controls (CTR) across three weight Sets (Set I, Set II, and Set III, based on animal body weight on day 0—the PH induction). AMB (10 mg/kg) and TAD (10 mg/kg) were administered by oral gavage once daily for 21 days, starting on day 8 after PH induction; veh groups received vehicle instead. Data are expressed as the means ± SEM; *n* = 3–10 rats per group. *^,∆,#^ *p* < 0.05; **^,∆∆^ *p* < 0.01; and ***^,∆∆∆^ *p* < 0.001—significant differences from * CTR + veh or ^∆^ MCT + veh within a given Set and from appropriate group in ^#^ Set I (200–219 g).

As shown in Figure 1, in MCT-induced PH rats, an enhancement of pressure in the pulmonary circulation was observed, as reflected by both the RVSP and mPAP. In comparison to the respective controls, RVSP (Figure 1a) was increased from about 25 mmHg to about 80 mmHg in Set I (+208%) and II (+219%) and to about 60 mmHg in Set III (+155%), whereas mPAP (Figure 1b) was elevated to about 40 mmHg in Set I (+110%) and II (+76%) and to about 30 mmHg (+37%) in Set III. Moreover, PH increased the Fulton’s index (Figure 1c) by 92%, 135%, and 77% in Set I, II, and III, respectively. Interestingly, only in Set II was the blood oxygen saturation (Figure 1d) significantly lower in PH animals (−13%). Treatment with AMB + TAD resulted in decreases in RVSP (−36% in Set I, −54% in Set II), mPAP (−23% in Set I, −28% in Set II), Fulton’s index (−26% in Set II), and an improvement in blood oxygen saturation (+15% in Set II).

Only mPAP demonstrated a strong negative correlation with initial BW in MCT + veh groups, whereas among treated individuals, blood oxygen saturation showed a moderate negative correlation with the initial BW (Table S1).

Survival rates (Figure 1e) in CTR + veh groups (all Sets) did not change over 4 weeks and remained at 100%. No deaths of animals were noted in any group until day 22. On day 29, mortality rates in PH animals reached the values of 67%, 22%, and 13% (in Set I, II, and III, respectively), whereas treated rats achieved the values of 38%, 38%, and 10% (in Set I, II, and III, respectively).

### 2.3. Influence of PH and Drug Therapy on RV and RA Hypertrophy

In order to evaluate if the whole right heart is affected by PH or the treatment in the initial BW-dependent manner, we performed complex morphometric assessments of both macroscopic RV and right atrium (RA) hypertrophy indices, as well as microscopic RV cardiomyocytes widths.

Similarly to Fulton’s index, the MCT increased other parameters of RV hypertrophy (RV weight to BW and RV weight to TL ratios) in all Sets of animals (+106 and +76%, respectively, in Set I; +181 and +139%, respectively, in Set II; +98 and +72%, respectively, in Set III). Only in Set II were similar increases in the whole heart weight (+38%), RV weight (+139%), and whole heart hypertrophy (expressed as heart weight/BW or heart weight/TL ratios) (+59 and +39%, respectively) noticed in PH animals. In other Sets, no changes were found or only tendencies were observed. Set II was also characterized by the strongest tendencies for increases in parameters related to the RA. Importantly, AMB + TAD diminished the MCT-induced RV hypertrophy: RV weight (−23%), and RV weight/TL ratio (−24%) in Set II only and tended to reduce the heart weight/BW ratio (Set II), RV weight (Set I), RV weight/BW (Set II), and RA-related parameters (Set I and Set II) (Table 1).

**Figure 2:**
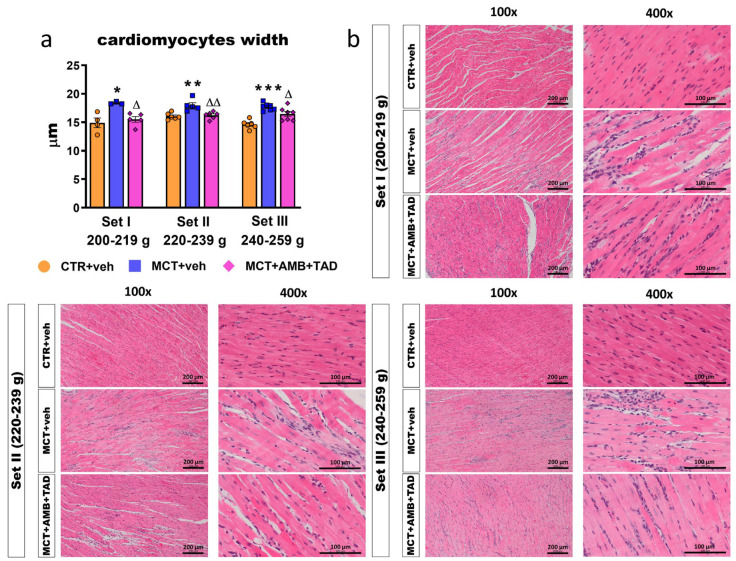
Influence of pulmonary hypertension (PH) and treatment with a combination of ambrisentan (AMB) and tadalafil (TAD) or their vehicle (veh) on right ventricle (RV) cardiomyocytes widths in monocrotaline (MCT)-induced PH rats and their controls (CTR) across three weight Sets (Set I, Set II, and Set III, based on animal body weight on day 0—the PH induction). AMB (10 mg/kg) and TAD (10 mg/kg) were administered by oral gavage once daily for 21 days, starting on day 8 after PH induction; veh groups received vehicle instead. The figure shows a column bar graph of RV cardiomyocytes widths (**a**) and representative hematoxylin and eosin-stained right ventricle images (100× and 400× magnification) (**b**). Data are expressed as the means ± SEM; *n* = 3–9 rats per group. *^,∆^ *p* < 0.05; **^,∆∆^ *p* < 0.01; and *** *p* < 0.001—significant differences from * CTR + veh or ^∆^ MCT + veh within a given Set.

As shown in Figure 2a, MCT increased the width of RV cardiomyocytes in all Sets of rats (+23% in Set I, +12% in Set II, and +21% in Set III). In all cases, pharmacotherapy reversed that PH-related effect (−16% in Set I, −10% in Set II, and −7% in Set III). Representative HE RV images (100× and 400× magnification) are shown in Figure 2b.

### 2.4. Influence of PH and Drug Therapy on RV Function

To determine whether not only the morphology but also the function of RV was affected by PH or treatment in the initial BW-dependent manner, we examined numerous hemodynamic parameters (via right heart catheterization and echocardiography).

The heart rate (HR) of animals measured by echocardiography on day 28 was significantly higher (in most cases) or tended to be higher (MCT and MCT + AMB + TAD groups in Set I) compared to the respective HR values measured during right heart catheterization on day 29. In Set II, MCT decreased the HR (−14%) compared to the control group, as measured by echo. A similar trend was observed in Set I (by echocardiography) and Set III (across both measurement methods). The AMB + TAD treatment did not significantly affect the HR in any of the Sets or measurement methods (Table 1).

Monocrotaline increased (in comparison to the respective controls) the rates of rise (dP/dt_max_) of RV pressure by 97, 100, and 83% in Sets I, II, and III, respectively (Table 1). In contrast, the rates of decrease (dP/dt_min_) of RV pressure were more negative by 90, 118, and 104% in Sets I, II, and III, respectively. The therapy partially reversed the above PH-related changes in Set I (by 28% in dP/dt_min_) and in Set II (by 33% and 39% in dP/dt_max_ and dP/dt_min_, respectively) and tended to revert changes in dP/dt_max_ in Set I and in both parameters in Set III (in all cases by about 20%).

**Figure 3:**
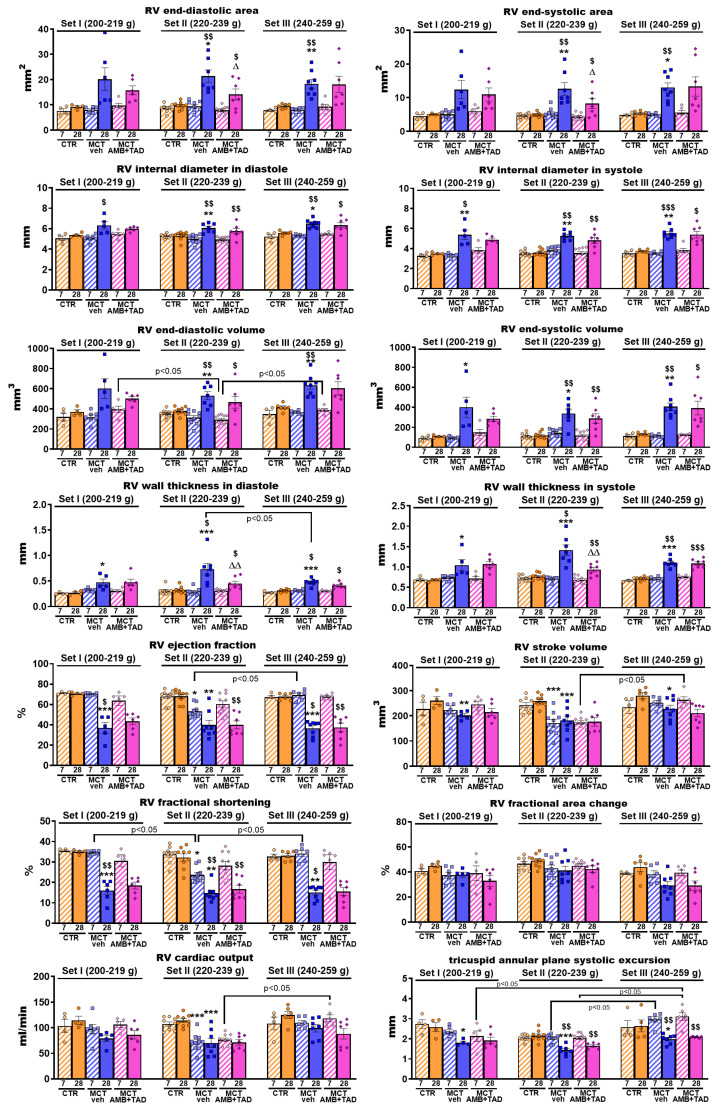
Influence of pulmonary hypertension (PH) and treatment with a combination of ambrisentan (AMB) and tadalafil (TAD) or their vehicle (veh) on right ventricular (RV) parameters measured by echocardiography in monocrotaline (MCT)-induced PH rats and their controls (CTR) across three weight Sets (Set I, Set II, and Set III, based on animal body weight on day 0—the PH induction). AMB (10 mg/kg) and TAD (10 mg/kg) were administered by oral gavage once daily for 21 days, starting on day 8 after PH induction; veh groups received vehicle instead. Echocardiographic measurements were performed on days 7 and 28. Data are expressed as the means ± SEM; *n* = 3–9 rats per group. *^,∆,$^ *p* < 0.05; **^,∆∆,$$^ *p* < 0.01; and ***^,$$$^ *p* < 0.001—significant differences from * CTR + veh or ^∆^ MCT + veh within a given Set and from ^$^ day 7. Differences between Sets are labeled directly for greater clarity.

Figure 3 shows the MCT-induced PH-related changes in cardiac parameters measured by echocardiography in three weight Sets at two time points (day 7 and day 28 after PH induction). No changes were observed in CTR groups, either between days 7 and 28 or between Sets. Only slight tendencies to increase the RV stroke volume, RV fractional area change, and RV cardiac output after 21 days of vehicle administration were noted. In most parameters there were no changes between experimental groups 7 days after MCT or its vehicle s.c. injection. However, in such a short time, the MCT managed to decrease RV stroke volume (−29%), RV ejection fraction (−21%), RV fractional shortening (−30%) and RV cardiac output (−33%) but only in Set II. Twenty eight days after MCT or its vehicle administration, PH-related changes were way more intense, i.e., MCT increased or tended to increase the RV end-diastolic (+119% in Set II; +92% in Set III) and end-systolic (+155% in Set II; +144% in Set III) areas, RV internal diameter in diastole (+15% in Set II; +16% in Set III) and systole (+54% in Set I; +47% in Set II; and +48% in Set III), RV end-diastolic (+40% in Set II; +53% in Set III) and end-systolic (+268% in Set I; +182% in Set II; and +204% in Set III) volume, RV wall thickness in diastole (+78% in Set I; +124% in Set II; and +53% in Set III) and systole (+55% in Set I; +85% in Set II; and +54% in Set III) and decreased or tended to decrease the RV ejection fraction (−47% in Set I; −42% in Set II; and −46% in Set III), RV stroke volume (−22% in Set I; −30% in Set II; and −19% in Set III), RV fractional shortening (−54% in Set I; −54% in Set II; and −54% in Set III) and RV fractional area change, RV cardiac output (−38% in Set II), and tricuspid annular plane systolic excursion (TAPSE) (−30% in Set I; −33% in Set II; and −25% in Set III), compared to the values in the CTR group. What is more, most of those worsened parameters were either significantly different from the day 7 values or showed at least a tendency to change. The 21-day treatment with AMB + TAD partially reversed only a few parameters, namely RV end-diastolic (−34%) and end-systolic (−34%) areas, as well as RV wall thickness both in diastole (−38%) and systole (−34%), but only in Set II. Non-significant tendencies of the treatment to reduce PH-related aggravations were noted in the RV end-diastolic area (Set I), RV end-diastolic (Set I) and end-systolic (Set I and II) volumes, as well as RV wall thickness in diastole (Set III).

To investigate whether the elementary function of RV was affected by PH or treatment in the initial BW-dependent manner, the contractile studies on isolated papillary muscles were performed.

**Figure 4:**
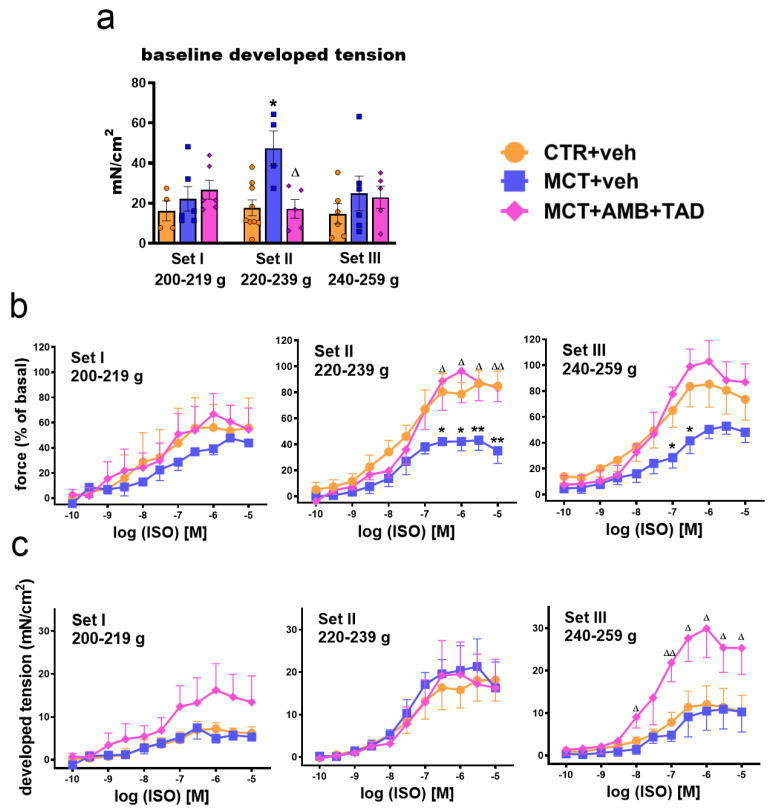
Influence of pulmonary hypertension (PH) and treatment with a combination of ambrisentan (AMB) and tadalafil (TAD) or their vehicle (veh) on function of right ventricular papillary muscles isolated from monocrotaline (MCT)-induced PH rats and their controls (CTR) across three weight Sets (Set I, Set II, and Set III, based on animal body weight on day 0—the PH induction). The figure shows baseline developed tension (**a**) and changes in force of contraction in response to increasing concentrations of isoprenaline (ISO; 0.0001–10 µM) expressed as percentages of basal values (**b**) and delta values from baseline developed tension (**c**). AMB (10 mg/kg) and TAD (10 mg/kg) were administered by oral gavage once daily for 21 days, starting on day 8 from PH induction; veh groups received vehicle instead. Data are expressed as the means ± SEM; *n* = 3–7 rats per group. *^,∆^ *p* < 0.05, **^,∆∆^ *p* < 0.01—significant differences from * CTR + veh or ^∆^ MCT + veh within a given Set.

In functional experiments, RV papillary muscles from MCT + veh animals (Set II) developed a higher baseline tension (+167%) compared to controls (Figure 4a), which was almost completely reversed after 21 days of treatment with AMB + TAD (−64%). The β-adrenoceptor agonist isoprenaline (ISO) (0.0001–10 μM) strengthened the contractions of papillary muscles in a concentration-dependent manner. Since the baseline developed tension differed, the inotropic effects of ISO were expressed both as percentages of basal values (Figure 4b, Table S2) and as changes in developed tension (delta) (Figure 4c, Table S2). Due to MCT-induced PH, only Set II showed a significant reduction in the maximal response (E_max_) to ISO (−50%), which was completely reversed with the AMB + TAD treatment (+123%) (Figure 4b, Table S2). However, the delta changes in developed tension were unaffected (Figure 4c, Table S2). Interestingly, in Sets I and III, the maximal response to ISO in the group that received MCT only tended to decrease (significantly in Set III at higher concentrations of 0.1–0.3 µM) in comparison to controls, but AMB + TAD treatment strongly enhanced (+87% and +173%) this response in Set III (Figure 4b,c, respectively; Table S2) and tended to do so in Set I. Both the PH and treatment did not alter the potency of ISO in all Sets (Figure 4b,c; Table S2).

### 2.5. Influence of PH and Drug Therapy on PA-Related Echocardiographic Parameters

In order to determine whether changes in PA structure and function were affected by PH or treatment in the initial BW-dependent manner, we performed echocardiographic examinations.

**Figure 5:**
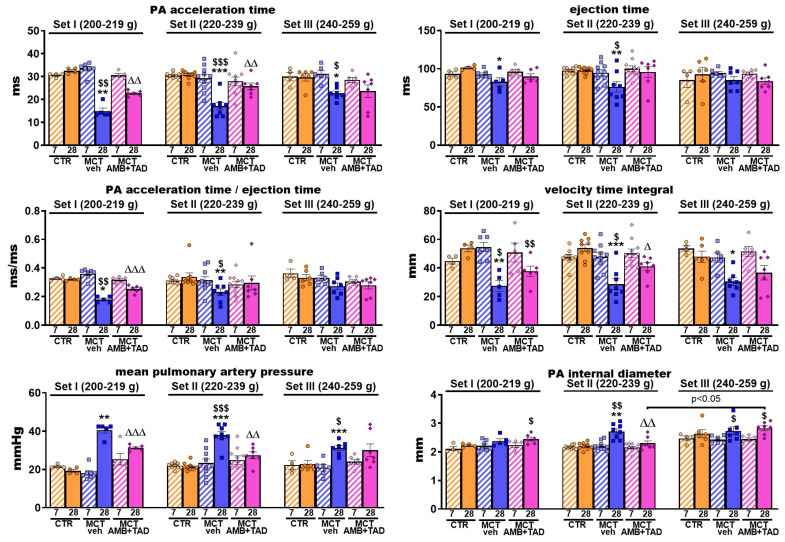
Influence of pulmonary hypertension (PH) and treatment with a combination of ambrisentan (AMB) and tadalafil (TAD) or their vehicle (veh) on echocardiographic pulmonary artery (PA) parameters in monocrotaline (MCT)-induced PH rats and their controls (CTR) across three weight Sets (Set I, Set II, and Set III, based on animal body weight on day 0—the PH induction). AMB (10 mg/kg) and TAD (10 mg/kg) were administered by oral gavage once daily for 21 days starting on day 8 from PH induction; veh groups received vehicle instead. Echocardiographic measurements were performed on days 7 and 28. Data are expressed as the means ± SEM; *n* = 3–9 rats per group. *^,∆,$^ *p* < 0.05; **^,∆∆,$$^ *p* < 0.01; and ***^,∆∆∆,$$$^ *p* < 0.001—significant differences from * CTR + veh or ^∆^ MCT + veh within a given Set and from ^$^ day 7. Differences between Sets are labeled directly for greater clarity.

Figure 5 shows MCT-induced, PH-related changes in PA parameters measured by echocardiography in three weight Sets at two time points (day 7 and day 28 after PH induction). No changes in CTR groups, as well as no intra- or inter-Set differences between experimental groups 7 days after MCT or its vehicle s.c. injection, were observed. On day 28, fully developed PH resulted in a decreased PA acceleration time (−54% in Set I; −44% in Set II; and −24% in Set III), ejection time (−18% in Set I; −22% in Set II), the ratio of the two above parameters (−44% in Set I; −32% in Set II; and tendency to fall in Set III), velocity time integral (−49% in Set I; −47% in Set II; and −36% in Set III), as well as in increased mPAP (+110% in Set I; +76% in Set II; and +37% in Set III) and the PA internal diameter (+23% in Set II). Treatment with AMB + TAD partially reversed PH-caused decreases in the PA acceleration time (+52% in Set I; +51% in Set II), PA acceleration time to ejection time ratio (+41% in Set I), and velocity time integral (+43% in Set II) and tended to increase the PA ejection time (Set II), PA acceleration time to ejection time ratio (Set II), and PA velocity time integral (Set I and III). Moreover, it effectively diminished the elevation of mPAP (−23% in Set I; −28% in Set II) and PA internal diameter (−15% in Set II) induced by MCT.

### 2.6. Influence of PH and Drug Therapy on Pulmonary Vascular Remodeling and Lung Hypertrophy

To establish whether hypertrophic changes in lungs were affected by PH or treatment in the initial BW-dependent manner, both lung hypertrophy and muscularization of the PA were determined.

**Figure 6:**
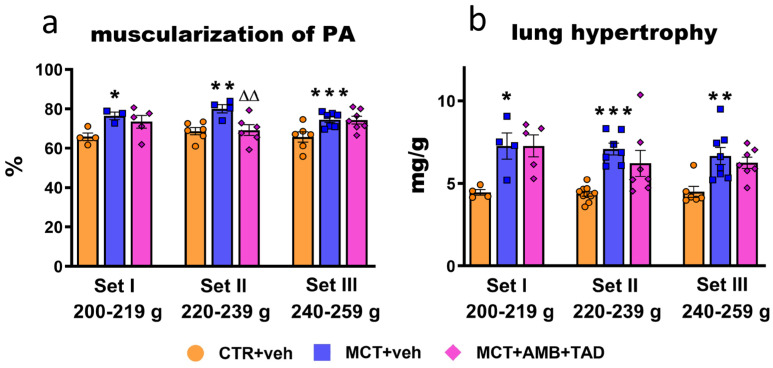
Influence of pulmonary hypertension (PH) and treatment with a combination of ambrisentan (AMB) and tadalafil (TAD) or their vehicle (veh) on lung parameters in monocrotaline (MCT)-induced PH rats and their controls (CTR) across three weight sets (Set I, Set II, and Set III, based on animal body weight on day 0—the PH induction). The figure shows the percentage muscularization of the pulmonary arteries (PA) (**a**) and lung hypertrophy expressed as lung weight to body weight ratio (**b**). AMB (10 mg/kg) and TAD (10 mg/kg) were administered by oral gavage once daily for 21 days, starting on day 8 from PH induction; veh groups received vehicle instead. Data are expressed as the means ± SEM; *n* = 3–10 rats per group. * *p* < 0.05; **^,∆∆^ *p* < 0.01; and *** *p* < 0.001—significant differences from * CTR + veh or ^∆^ MCT + veh within a given Set.

As shown in Figure 6, MCT caused significant changes in lung tissue. The development of PH was accompanied by the increased muscularization of pulmonary arteries (PA) (+16% in Set I; +17% in Set II; and +13% in Set III) (Figure 6a) and lung hypertrophy (+63% in Set I; +62% in Set II; and +48% in Set III) (Figure 6b). The treatment with dual therapy decreased (−14%) muscularization of the PA and only tended to lower lung hypertrophy in Set II of the animals. Representative HE pulmonary images (100× and 400× magnification) are shown in Figure S1.

### 2.7. Influence of PH and Drug Therapy on Isolated PA Functional Studies

To find out whether PH or therapy can alter other aspects of PA function in the initial BW-dependent manner, we performed reactivity experiments on isolated PAs.

**Figure 7:**
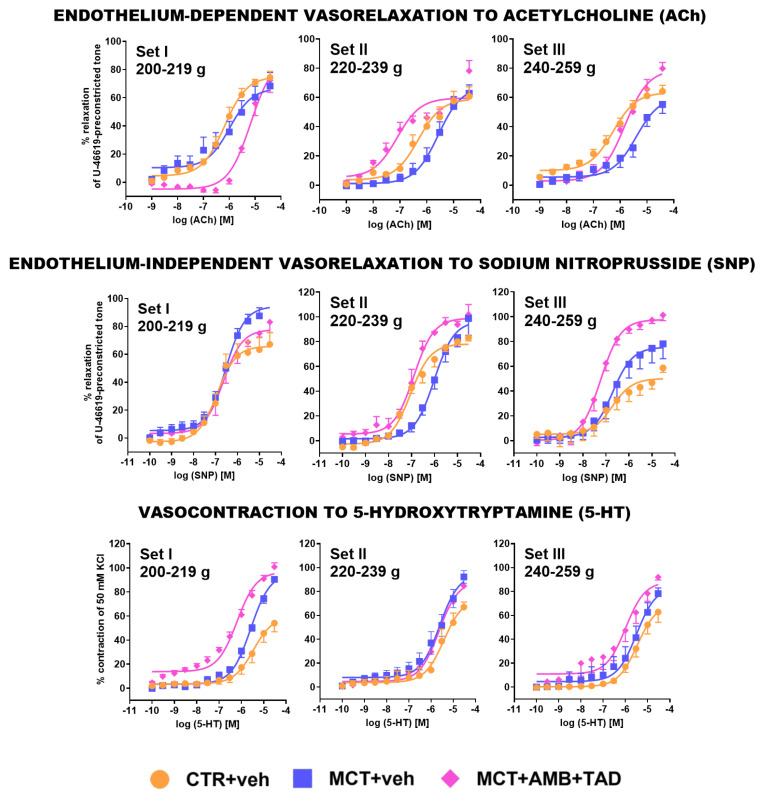
Influence of pulmonary hypertension (PH) and treatment with a combination of ambrisentan (AMB) and tadalafil (TAD) or their vehicle (veh) on responses to acetylcholine, sodium nitroprusside, and 5-hydroxytryptamine in pulmonary arteries isolated form monocrotaline (MCT)-induced PH rats and their controls (CTR) across three weight Sets (Set I, Set II, and Set III, based on animal body weight on day 0—the PH induction). AMB (10 mg/kg) and TAD (10 mg/kg) were administered by oral gavage once daily for 21 days starting on day 8 from PH induction; veh groups received vehicle instead. Data are expressed as the means ± SEM; *n* = 3–8 rats per group.

Figure 7 and Table S3 show changes in the reactivity of PAs in response to different relaxing agents, i.e., endothelium-dependent acetylcholine (ACh) and endothelium-independent sodium nitroprusside (SNP) or constricting 5-hydroxytryptamine (5-HT). Acetylcholine (0.001−30 μM) and SNP (0.0001−30 μM) produced a concentration-dependent relaxation in the thromboxane A_2_ analog U-46619-pre-constricted tone, and 5-HT (0.0001−30 μM) induced a concentration-dependent contraction of the isolated PAs in all experimental groups. Responses to all studied agonists were equipotent in the respective control groups in each Set, except for the reduced E_max_ obtained by SNP in Set III, when compared with Set II (−29%).

Pulmonary hypertension lowered the potency of ACh in Set II (−11%) and Set III (−16%) but not in Set I, as well as the potency of SNP in Set I (−6%) and Set II (−14%) but not in Set III (Figure 7, Table S3). The efficacy of ACh was not affected by MCT significantly; however, in the case of SNP, E_max_ was increased in Set I (+49%) and tended to do so in Set II. MCT did not affect the pEC_50_ of 5-HT, but it did increase the efficacy of 5-HT in Set I (+67%) and Set II (+38%) and tended to increase it in Set III.

Treatment with AMB + TAD decreased the pEC_50_ of ACh (−15%) and tended to decrease the E_max_ of SNP in Set I, but increased (in comparison to the MCT-treated group) the potency of both ACh and SNP in Set II (+27% and +15%, respectively) and Set III (+7% and +7%, respectively) and increased the efficacy of ACh (+45%) and SNP (+73%) in Set III, as well as tended to increase the E_max_ of ACh in Set II. Moreover, AMB + TAD increased the potency of 5-HT in Set I (+11%) and III (+11%).

### 2.8. Influence of PH and Drug Therapy on Parameters Not Related Directly to PH

To determine if the initial BW-dependent changes in the PH and treated groups are specific to the right heart, we examined left-sided echocardiographic and morphometrical parameters, as well as measured basic blood parameters and kidney weight.

The left-sided changes in echocardiographic parameters were summarized in Table S4. No differences were noted on day 7 after MCT injection and among CTR groups. The full course of MCT-induced PH caused a decrease in LV stroke volume (−31% in Set I; −37% in Set II; and −26% in Set III), LV ejection fraction (−22% in Set I; −20% in Set III), LV fractional shortening (−29% in Set I; −35% in Set III), and LV cardiac output (−38% in Set I; −46% in Set II; and −27% in Set III), compared to controls. No effects of treatment were noticed.

Monocrotaline also did not change parameters related to the left side of the heart, except for an increase in LV with the septum (LV + S) to BW ratio (+18%) in Set II. Moreover, it did not modify kidney hypertrophy parameters and glucose, triglycerides, cholesterol, or lactate levels in any Set or group. However, MCT tended to slightly decrease the size of the kidney and the level of blood glucose or triglycerides and to increase level of lactates vs. controls (Table S5).

## 3. Discussion

### 3.1. General

The aim of our study was to examine the influence of rats’ initial BWs on the severity of PH and on the effectiveness of the reference therapy. Many factors affecting PH severity, as well as the therapeutic potency of investigated drugs, have been identified (see Section 1). However, the initial BW was not thoroughly examined yet. In our previous studies, we observed that even a few weeks’ variation in the rat’s age can lead to differing outcomes both in the animal models of systemic hypertension [26] and experimental PH [27,28]. In the current experiments, we induced PH by a single s.c. injection of MCT (60 mg/kg) in male Wistar rats, which is one of the most frequent and one of the two ‘classical’ experimental models of this disease, alongside chronic hypoxia. We have decided to verify our hypothesis using the MCT model due to its simplicity, reproducibility, relatively low cost, and, consequently, wide applicability, which allows for a broader discussion and comparison with the results obtained by other authors. Moreover, as mentioned above, our own experience suggests a relationship between the initial BW and development of PH in this particular model. According to the systematic review and meta-analysis by Sztuka and Jasińska-Stroschein [13], based on data from 291 studies on animal models of PH, involving a total number of 6126 animals, most experiments were performed on rats (92.96%), predominantly Sprague Dawley (57.28%) and Wistar (34.53%). Additionally, 76% of interventions were based on a single MCT injection at a dose of 60 mg/kg, and male animals were used in 94.92% of the cases. Similarly, the analysis of the data from 409 animal studies toward better reproducibility in experimental research on new agents for PH demonstrated that, in the majority of the above studies, the most common model was based on a single MCT injection (58.5%) at a dose of 60 mg/kg or more (48.2%) [16]. Due to its rapid metabolism in mice, MCT is administered predominantly to rats [9].

The female sex is a known risk factor for PAH, with a higher female-to-male ratio observed in individuals affected by this condition. However, women with PAH tend to have a better RV function and a more favorable prognosis than their male counterparts. Such a phenomenon was described as the ‘estrogen paradox’ [21,29,30,31]. In contrast, in preclinical studies, the development of MCT-induced PH is less pronounced in females than in male animals [25,32]. Therefore, we used male rats only, which stays in line with the majority of previously conducted experiments [13,16].

As the reference therapy, we applied the combination of the inhibitor of phosphodiesterase 5 (PDE-5) tadalafil (TAD) together with the endothelin 1 (ET-1) receptor antagonist ambrisentan (AMB), since the early combination therapy is currently the gold standard for most patients with PAH [5]. Its clinical effectiveness and superiority over monotherapy were confirmed in the Ambrisentan and Tadalafil in Patients with Pulmonary Arterial Hypertension (AMBITION) trial [33]. In our experiments, ambrisentan and tadalafil (AMB + TAD) were administered orally at doses of 10 mg/kg each for three weeks. This treatment regimen was chosen because it has been shown to be more effective than monotherapy in treating experimental PH in rats [34]. Additionally, monotherapies of the same doses of AMB [35] and TAD [36,37,38,39], as well as the combination of TAD plus the other ET-1 receptor antagonist, macitentan [36], have also been proven to be effective in experimental PH. We administered both compounds in a therapeutic regimen, as it better reflects PAH therapy in humans, in contrast to the more commonly used preventive regimen [16]. We were unable to start therapy later than one week after MCT administration and had to limit the treatment duration to 21 days due to increased rat mortality observed around 22–24 days after MCT injection (Figure 1).

The choice of ranges for the initial BW: 200–219 g (Set I), 220–239 g (Set II), and 240–259 g (Set III) was based both on the literature data with similar experimental conditions (MCT at 60 mg/kg in Wistar rats) (for details, see Section 3.2) and our previous experience.

### 3.2. Initial Rat BW Affects the Severity of MCT-Induced PH

To provide a clearer and more concise overview of the changes in numerous parameters assessed under the current study (Figure 1, Figure 2, Figure 3, Figure 4, Figure 5, Figure 6 and Figure 7; Table 1, Tables S2 and S3), we have summarized in Table 2 their direction and percentage changes. The development of MCT-PH was evidenced by a wide range of parameters, including mPAP (the primary diagnostic criterion for PAH in humans), RVSP, and Fulton’s index. Importantly, we observed that the initial BW influenced the degree of these changes. mPAP, RVSP, and Fulton’s index tended to be higher in Set I (200–219 g) and Set II (220–239 g) compared to Set III (240–260 g). At the same time, the most aggressive PH development in Set I (200–219 g) was associated with a very high mortality rate (approximately 70%). A decrease in blood oxygen saturation was noted only in Set II. What is more, only mPAP demonstrated a negative correlation with the initial BW, which indicates a lack or weaker linear association among other parameters. Importantly, it is worth noting that the lack of linearity in the correlation between the initial BW and parameters related to the severity of PH may be largely due to the high mortality rate in Set I. We can assume that, if not for premature death, individuals in this Set could have been characterized by the highest values of the mPAP, RVSP, Fulton index, and the lowest blood oxygen saturation.

We noticed high RVSP values—about 80 mmHg in Sets I and II and approximately 60 mmHg in Set III. Similar increases in RVSP were observed in other publications using male Wistar rats of an initial BW of (1) 200–220 g, (2) 240–260 g, and (3) over 300 g. Thus, 4 weeks after MCT administration at 60 mg/kg, the RVSP (1) reached 70–80 mmHg under isoflurane [40] or ketamine/xylazine [41] anesthesia; (2) around 60 mmHg under tribromoethanol anesthesia [42]; and (3) about 30–50 mmHg under ketamine/xylazine [27] or isoflurane [43,44,45,46] anesthesia, respectively. In contrast, in another study, lower RVSP values were observed in animals weighing 200–220 g, i.e., 40–45 mmHg under isoflurane anesthesia [47]. Notably, isoflurane or ketamine/xylazine anesthesia can itself elevate this parameter [13]. A relatively high mortality rate (around 40%) was reported by Souza et al. [40] and Silva et al. [48] in experiments with rats of an initial BW around 180–240 g. Similarly, Kawade et al. [49] demonstrated a higher survival rate and less severe PH after MCT (60 mg/kg) in 20-week-old than in 7-week-old Sprague-Dawley rats.

Four weeks after MCT administration, the progression of PH led to significant cardiac hypertrophy. This was evidenced by an increase in the Fulton’s index, RV weight (expressed as absolute values [in Set II only] and as ratios to BW or TL [in all Sets]), and in RV cardiomyocyte (in a comparable way in all Sets). Notably, the increases in the ratios of RV to BW and TL tended to be the highest in Set II (Table 2). Echocardiographic examination further confirmed RV hypertrophy, showing increased RV wall thickness in both diastole and systole (the highest values were obtained in Set II). In contrast, hypertrophy of the RA was less pronounced, with a more apparent trend seen in Set II only.

Right ventricular hypertrophy was associated with changes in cardiac function. Basal HR was higher in animals anesthetized with isoflurane (echocardiography) compared to ketamine/xylazine (right heart catheterization). That could likely be because of the cardiodepressive effect of ketamine/xylazine anesthesia [50]. The higher basal values might explain why a decrease in HR was observed in MCT-treated rats during echocardiographic evaluation only (in Set II exclusively). Moreover, hypertrophy of the right heart in PH has been shown to impair the sinus node function, leading to a decrease in HR [51]. MCT-induced bradycardia, as well as higher rates of rise (dP/dt_max_) and more negative rates of decrease (dP/dt_min_) in RV pressure (comparable in all three Sets), has been previously observed in severe PH [52,53].

Developing PH also caused alterations in other echocardiographic parameters. The first differences were already noticed 7 days post-MCT injection (i.e., decreases in RV stroke volume, RV ejection fraction, RV fractional shortening, and RV cardiac output) but only in Set II. Changes in all echocardiographic parameters, which were partially influenced by the initial BW, occurred 4 weeks after MCT administration. At that point, we observed typical increases in RV end-diastolic and end-systolic areas (Sets II and III, but with RV fractional area change unaltered) and in the RV internal diameter during both diastole and systole, along with an increase in RV end-systolic and end-diastolic volumes (Table 2). However, in Set I, only slight tendencies towards enhancement in RV end-systolic and end-diastolic areas, as well as RV end-diastolic volume, were noticed. Additionally, an elevated afterload led to RV stroke volume and ejection fraction deterioration across all sets. At the same time, a significant decrease in cardiac output was observed in Set II only, likely due to HR reduction, which was also only seen in Set II. Moreover, the tricuspid annular plane systolic excursion (TAPSE), a measure of RV systolic function, was significantly impaired across all Sets. Similar changes in the above echocardiographic parameters have been previously reported in MCT-induced PH rats [14,44,46,53].

Pulmonary hypertension, by increasing sympathetic activity, alters β-adrenergic receptor responsiveness in the RV. However, the dynamics of these changes remain less examined compared to analogous changes observed in LV hypertrophy [54]. To address this gap, and to account for the potential influence of the rats’ BWs, we investigated the functional response to β-adrenergic receptor stimulation in isolated RV papillary muscles. We demonstrated that in Set II (but not in Sets I and III), the muscle generated, developing tension at the baseline in PH rats, was significantly higher than in controls. Note that, at the same time, RV hypertrophy tended to be higher in Set II than in I and III. Similar increases in developed tension at the baseline have been reported previously in RV trabeculae and papillary muscles isolated from MCT-PH rats and explained both by RV hypertrophy and enhanced Ca^2+^ responsiveness observed in MCT hearts [55,56]. Differences in the baseline developed tension were the reason that we expressed inotropic effects of isoprenaline in RV papillary muscles both as a percentage of basal and as a delta change in the force of contraction. However, the papillary muscles of MCT-treated rats showed a reduction in the maximum response to isoprenaline (represented as a percentage of basal) in Set II, with slight tendencies in Set I and III but not when expressed as delta changes. Kögler et al. [56] demonstrated that the last parameter represents the reduced contractile reserve of MCT cardiac papillary muscles (after β-adrenergic stimulation), even despite an increased baseline developed tension. The decrease in the inotropic effect of isoprenaline might be related to RV-specific desensitization to β-adrenergic stimulation, changes in Ca^2+^ cycling, reductions in cAMP, and alterations in noradrenaline uptake observed in cardiomyocytes isolated from rats with PH [25,57].

In contrast to evident MCT-induced morphological and functional abnormalities affecting the RV, we noticed only a few changes in the LV. Thus, LV hypertrophy was confirmed only in Set II, in which we determined a greater LV + S weight to BW ratio in comparison to respective controls. In addition, the echocardiographic examination showed significant decreases in the LV stroke volume and cardiac output (all Sets) and in LV ejection fraction and fractional shortening (Sets I and III). A detailed analysis of 135 studies and 207 interventions by Jasińska-Stroschein [14] demonstrated that only MCT-induced PH (but not other experimental PH models) might cause significant LV hypertrophy, especially in the case of experiments lasting 4 weeks or more. Moreover, exaggerated PH was correlated with systolic dysfunction featured by a decrease in the LV ejection fraction.

As summarized in Table 2, we also observed a partial influence of the initial BW on PH-related changes in pulmonary tissue (Figure 6). Firstly, MCT caused lung hypertrophy and increased muscularization of the PA, which was comparable between Sets. Secondly, mPAP and other functional parameters measured by echocardiography, such as the PA acceleration time, PA ejection time, PA AT/ET ratio, and velocity time integral, were comparable between Sets I and II, with Set III being the least affected. Additionally, the PA internal diameter was increased in Set II only (Table 2). The above directions of change (increase or decrease) are typical for MCT-induced PH [14,44,46,53].

Ultimately, we assessed PA reactivity to the endothelium-dependent and endothelium-independent vasorelaxants ACh and SNP, respectively, as well as to the vasoconstrictors 5-HT. MCT led to a decrease in the affinity (but not efficacy) of ACh (Sets II and III only). In the case of SNP, it reduced the affinity while increasing efficacy (in Set I). Moreover, it enhanced the efficacy of 5-HT in Sets I and II. Thus, we observed the most significant modulations in Set II, and we confirmed endothelial dysfunction in PAs. The lack of differences in the maximal effects of vasorelaxants might be attributed to moderate muscularization of the vascular smooth muscles observed in histological settings. Our functional findings align, at least partially, with previous reports in similar experimental settings, which also noted a reduction in ACh and/or SNP affinity [28,58,59] and an enhanced vasoconstrictor response to 5-HT [60,61]. In contrast to our results, in the latter studies, MCT-treatment also affected the efficacy of ACh and SNP [28,59]. The observed differences might result from species-specific variations: (1) Wistar [28,58,59,60] vs. Sprague Dawley rats [61]; (2) the dose of MCT used to induce PH: 60 mg/kg [28,58,59,61,62] vs. 105 mg/kg [60]; (3) the duration of experimental protocol (time post-MCT injection): 21 days [28,58,61] vs. 24 days [59]; (4) differences in the morphometric and functional characteristics of the pulmonary artery: main [58,60,61] vs. intrapulmonary artery [28,59]; (5) other BW ranges: 180–200 g [59]; 150–250 g [28]; and 250–300 g [61]; or (6) other experimental conditions.

Overall, our study is the first to clearly demonstrate the significant influence of even relatively small differences in initial BW on the development of PH induced by MCT in male Wistar rats. As listed in Table 2, we determined the weight-dependent differences in various parameters, determining mainly RV cardiac morphology and function, as well as PA function under in vivo and ex vivo conditions. The ratio of the number of parameters in which the significant changes were noticed in Sets I, II, and III was 26:41:26, respectively (Table 2). However, one should keep in mind that the number of significant changes in Set I could have been higher, but severe PH in this Set led to the highest mortality rate. Importantly, as mentioned above, similar observations were confirmed in other studies in MCT-treated rats that differed in initial BW. However, only in the current study were all other factors that can affect the severity of PH development (listed in the Section 1) excluded, since all experiments were performed under exactly the same conditions. Moreover, our results are in line with the so-called ‘obesity paradox’, according to which, in many cardiopulmonary conditions, including PAH, high BW and/or body mass index are associated with the increased survival of patients [63,64].

### 3.3. Initial Rat BW Affects the Effectiveness of the Reference Therapy in MCT-Induced PH Model

As summarized in Table 2, under in vivo conditions, a 3-week combined reference therapy with AMB + TAD in a therapeutic regimen appeared to be the most beneficial in Set II. Plenty of critical parameters were improved, including mPAP, RVSP, and blood oxygen saturation, as well as cardiac hypertrophy (Fulton’s index, RV weight, and RV wall thickness in diastole and systole). The correlation analyses between the initial BW and the most crucial PH indicators, showing only moderate linearity in one parameter (SpO_2_) after treatment, may be due to the fact that mostly non-linear correlations were identified in the MCT + veh group (for rationale, see Section 3.2). Certain cardiac function parameters (dP/dt_max_ and dP/dt_min_, RV end-diastolic and end-systolic areas) were also positively affected but predominantly in Set II. Moreover, the therapy tended to reduce lung hypertrophy and exerted a beneficial influence on PA muscularization, along with improvements in PA functional parameters (PA acceleration time and velocity time integral) but, again, mostly in Set II. The RV cardiomyocyte width was diminished under therapy in all Sets. Unfortunately, therapy failed to improve the mortality rate.

The combined therapy also affected the contractility of RV papillary muscles and the vasoreactivity of PA under ex vivo conditions. Specifically, in cardiac tissue, it prevented the enhancement of baseline developed tension (in Set II only) and increased the inotropic effect of isoprenaline (Sets II and III). This seems an interesting observation, since the data regarding the influence of AMB + TAD on RV papillary muscles is limited, even when administered as a monotherapy despite both ET-1 receptors and PDE-5 being expressed in the rat heart [65,66]. Our results may, therefore, suggest that AMB + TAD have a direct cardioprotective effect in PH.

In PAs isolated from MCT-induced PH rats, chronic administration of combined pulmonary vasodilator therapy (AMB + TAD) significantly enhanced both endothelium-dependent vasorelaxant responses to ACh and endothelium-independent vasorelaxation to SNP in Sets II and III. This stands in line with previous findings showing that chronic AMB + TAD treatment increased small vessel volume density in female MCT-treated rats [67], and when administered acutely, it acted synergistically to relax ET-1-constricted rat PA preparations [68]. However, unexpectedly, the reference therapy increased the maximal response to 5-HT in Sets I and III. We hypothesize that this phenomenon might be due to a shared signaling pathway or an interaction between 5-HT and ET-1 [69]. It is plausible that when one vasoconstrictive pathway, such as the ET-1-mediated pathway, is inhibited, the alternative ones, including 5-HT, become upregulated, which results in a compensatory vasocontractile response.

The combination therapy of AMB + TAD has become the gold standard for treating PAH in humans [5], primarily acting as a vasodilator and exhibiting antiproliferative effects. Our results further confirm the beneficial impact of such a treatment on PAs (demonstrating that vascular benefits are primarily driven by promoting vasorelaxation rather than suppressing vasoconstriction) and on cardiac hypertrophy. Only three studies have reported the positive effects of chronic AMB + TAD administration in experimental PH models so far, including MCT-induced PH (45 mg/kg) combined with left pneumonectomy [67] and Sugen/hypoxia-induced PH [34,35] in female and male Sprague Dawley rats, respectively. The authors of the above papers noticed similar findings to our results, demonstrating improvements in the mPAP, RVSP, Fulton’s index, blood oxygen saturation, and relative vessel wall thickness [34,35,67]. However, as some animals in these studies died, the authors suggested the addition of a third drug [67].

To the best of our knowledge, the current study is the first to demonstrate the influence of even relatively small differences in initial BW on the effectiveness of reference therapy against PH induced by MCT in male Wistar rats. The ratio of the number of parameters in which the significant changes were noticed in Sets I, II, and III was 8:22:10, respectively, (Table 2).

### 3.4. Limitations of the Study

In the present study, we determined the significant impact of relatively small differences in initial BW on (1) the severity of PH development and (2) the effectiveness of the chosen reference therapy in MCT-induced PH in male Wistar rats. Other factors affecting these two points have been described in detail in previous studies [13,14]. Thus, one should keep in mind that different results may be obtained if (1) the other experimental model of PH is used, e.g., Sugen/hypoxia; (2) a different time frame of PH development following induction is applied; (3) female animals are used; (4) a different rat strain is employed; (5) another weight range is considered, e.g., below 200 g, between 200 and 300 g, and above 300 g; or (6) a larger sample size is introduced (especially in groups with a high mortality).

## 4. Materials and Methods

### 4.1. Animals

All procedures and experimental protocols were performed in accordance with the ARRIVE guidelines, European Directive (2010/63/EU), and under the approval of the local Animal Ethics Committee in Olsztyn (Poland) (approval code 5/2022). Wistar rats were acquired from the Centre for Experimental Medicine at the Medical University of Bialystok (Poland). They had ad libitum access to food and water and were housed under a 12:12 h light–dark cycle with a constant temperature (21 ± 2 °C) and humidity (55 ± 5%).

### 4.2. Protocol and Experimental Groups

On day 0, 72 male Wistar rats (6–8 weeks old) were divided, based on their body weight (BW), into three Sets: Set I (initial BW 200–219 g), Set II (initial BW 220–239 g), and Set III (initial BW 240–259 g). Only animals that fell within one of the three BW ranges on day 0 were included in the study. After the animals were assigned, they were given a single subcutaneous (s.c.) injection of monocrotaline (MCT) (60 mg/kg in a volume of 3 mL/kg) to induce pulmonary hypertension. Identical and contemporaneous controls (CTR) received a s.c. injection of an equal volume of 0.9% NaCl [27].

Starting from day 8, the combination of ambrisentan (AMB) (10 mg/kg) and tadalafil (TAD) (10 mg/kg) was given to MCT rats, whereas vehicle for AMB and TAD (veh; 0.5% hydroxypropyl methylcellulose, 4 mL/kg) was administered to MCT and CTR rats by oral gavage every 24 h for a period of 21 days. Thus, in each Set, there were three following groups: (1) CTR + veh; (2) MCT + veh; and (3) MCT + AMB + TAD. The assignment of animals to the above groups and cage location, as well as the order of the treatment and measurements, were random. No a priori exclusion criteria were established. Only the principal investigator knew the exact allocation of animals to groups during the experiments. Due to differences in the appearance of the administered compounds, the individuals administering them were partially aware of the allocation. All investigators were aware of the animal allocation during the statistical analysis.

### 4.3. Echocardiographic Measurements

Echocardiographic imaging was performed using the Alpinion ECUBE 15 Platinum ultrasound system (Alpinion Medical Systems, Seoul, Republic of Korea) on day 7 and day 28 of experimental protocol, i.e., 24 h before the start and at the endpoint of applied treatment. After the onset of anesthesia with 2.5% isoflurane in an induction chamber (SomnoSuite^®^ Low-Flow Anesthesia System, Kent Scientific Corporation, Torrington, CT, USA; average gas flow 180–250 mL/min for animals of BW 200–300 g and 250–300 mL/min for animals of BW 300–400 g), rats were immediately transferred to a low-profile mask for anesthesia maintenance and simultaneously placed in a supine position on a heating pad. The body temperature was kept at 37 ± 0.5 °C. After shaving the thorax, hair removal cream was applied. Then, after placing ultrasound transmission gel on a probe, series of measurements were performed using a 17 MHz linear transducer. Complete 2-Dimensional (2-D), M-Mode, and Pulsed-Wave (PW) Doppler echocardiograms were collected. Then, the images were digitally stored for further analysis.

Right ventricular (RV) and left ventricular (LV) end-diastolic (EDA) and end-systolic (ESA) areas, end-diastolic (IDD) and end-systolic (IDS) internal diameters, and LV wall thickness in end-diastole (LVWTd) and end-systole (LVWTs) were measured using M-mode, parasternal short-axis (SAX) view, whereas RV wall thickness in diastole (RVWTd) and systole (RVWTs) was measured using M-mode, parasternal long-axis (LAX) view. Pulmonary artery (PA) diameter was measured by placing the probe in a superior angulation of a parasternal SAX position to visualize RV outflow tract (RVOT). Pulsed-Wave Doppler measurements were then performed, and PA acceleration time (PAAT), pulmonary ejection time (ET), PAAT/ET ratio, and velocity time integral (VTI) were subsequently calculated. Tricuspid annular plane systolic excursion (TAPSE) was measured in apical four-chamber view. End-diastolic (EDV) and end-systolic (ESV) volumes of both LV and RV were estimated using the Teichholz formula, i.e., 7 × D^3^/(2.4 + D); D—internal diameter of a given ventricle in diastole/systole. Stroke volume (SV) equals the difference between EDV and ESV (SV = EDV − ESV). Cardiac output (CO) equals the SV multiplied by heart rate (HR) for the respective ventricle: CO = SV × HR. Ejection fractions (EF) were calculated using the formula EF = EDV − ESV/EDV × 100%. Similarly, fractional shortening (FS) values were obtained using the formula FS = IDD − IDS/IDD × 100% and fractional area changes (FAC) by applying the formula FAC = EDA − ESA/EDA × 100%. Mean pulmonary artery pressure (mPAP) was calculated according to Urboniene et al. [70], using the formula mPAP = 58.7 − 1.21 × PAAT. The collection of data was conducted by researchers unaware of the outcomes from other modalities. Consequently, gathered images were analyzed by one investigator to exclude interobserver variability. Each examination typically lasted for no longer than 20–25 min; following that, rats were given time to recover from the procedure.

### 4.4. Determination of Blood Oxygen Saturation

Blood oxygen saturation was measured on day 28 during echocardiography using a pulse oximeter (MouseSTAT^®^ Jr Rodent Pulse Oximeter and Heart Rate Monitor with Rat Paw Pulse Oximeter Sensor, Kent Scientific Corporation, Torrington, CT, USA). The device was attached to the left hind paw of the animal immediately after isoflurane anesthesia induction.

### 4.5. Determination of Parameters in Tail-Tip Blood Samples

Blood glucose, cholesterol, triglycerides, and lactate levels were measured on day 29, 24 h after the treatment endpoint, right before ketamine/xylazine anesthesia, in tail-tip blood samples, using the Accu-Chek blood glucose meter (Roche, Basel, Switzerland) and Accu-Trend Plus system (Roche, Basel, Switzerland) with appropriate disposable measuring strips. The tip of the rat tail, locally anesthetized with 10% lidocaine spray, was cut off, and then blood samples were collected into heparinized capillaries.

### 4.6. Determination of Right Ventricular Systolic Pressure

After anesthesia was induced with ketamine and xylazine (intraperitoneally, ca. 109 mg + 2.2 mg/kg BW, respectively; 1.2 mL/kg BW)*,* a pressure sensor-equipped catheter (SPR-320 Mikro-Tip, Millar, Pearland, TX, USA) was pushed forward through the right jugular vein and positioned in the RV. Then, RV systolic pressure (RVSP), HR, and rate of rise/decrease in RV pressure (dP/dt_min/max_) were measured. Data were collected using LabChart 7.3.7 Pro (ADInstruments, Dunedin, New Zealand).

### 4.7. Determination of Organ Weight and Hypertrophy Indices

After RVSP determination, the heart, lungs, kidneys, and tibia (from right hind paw) were removed. The RV, LV with septum (LV + S), right (RA) and left (LA) atria, lungs, and left kidney were then separated and weighed. RV hypertrophy was expressed in three ways: as Fulton’s index, which is the ratio of RV weight to LV + S (RV/LV + S), and as other hypertrophy indices, including the RV weight to BW ratio and the RV weight to tibia length (TL) ratio. The lung hypertrophy index was expressed as lung weight to BW ratio, whereas hypertrophy indices for LV, RA, LA, and the kidney were calculated as each organ’s weight relative to both BW and TL.

### 4.8. Functional Studies on Isolated Papillary Muscles

The papillary muscles were carefully separated from the RV and then mounted in 10 mL chambers of an organ bath and suspended vertically on isometric force transducers (FT20, HSE, March-Hugstetten, Germany), with tension set at 5 mN [71]. The preparations were electrically simulated using platinum electrodes (just over the threshold, 5 ms duration, 2.5 Hz) and stabilized in the organ bath containing Tyrode’s solution (mM): NaCl 119.8, KCl 5.45, MgCl_2_ 1.05, NaHCO_3_ 22.6, NaH_2_PO_4_ × H_2_O 0.42, CaCl_2_ × 2H_2_O 1.8, glucose 5.05, ascorbic acid 0.25, and EDTA 0.05 (pH 7.4; 37 °C) and gassed with carbogen (95% O_2_ and 5% CO_2_) for 90 min. Then, papillary muscles were exposed to increasing concentrations of isoprenaline (ISO; 0.0001–10 μM), a non-selective β-adrenoreceptor agonist, and concentration-response curves (CRCs) were subsequently generated. Muscle force was normalized to the cross-sectional area, calculated on the basis of the tissue’s length and weight, assuming a muscle density of 1.06 g/cm^3^ [72]. Data were collected by the data acquisition system (ADInstruments, Dunedin, New Zealand).

### 4.9. Preparation of Pulmonary Arteries

Rat PAs preparation and experimental procedures have been described in detail previously [28]. Following sacrifice, PAs were isolated from segmental branches, cleaned carefully, and cut into rings (2 mm in length and ~150 μm in internal diameter) from the middle portion of each artery. The arterial rings were mounted on stainless steel wires (Mulvany–Halpern-type wire myograph, model 620M; Danish Myo Technology, Hinnerup, Denmark) in 5 mL organ baths containing Krebs–Henseleit solution with the following composition (in mM): NaCl 118; KCl 4.8; CaCl_2_ 2.5; MgSO_4_ 1.2; NaHCO_3_ 24; KH_2_PO_4_ 1.2; glucose 11; and EDTA 0.03. The baths were continuously gassed with 95% O_2_ and 5% CO_2_ at 37 °C (pH 7.4). PA rings were set at tensions corresponding to their mean in vivo RV pressure using the Laplace equation and then were allowed to equilibrate for 30 min. Isometric muscle tension was measured and recorded using LabChart 8.1.27 Pro (ADInstruments, Dunedin, New Zealand). To test vascular viability, the rings were exposed twice to KCl (50 mM). Vessels that constricted ≥ 1 mN were subsequently used. After washing out, the functional state of the endothelium was assessed by evaluating the response to muscarinic receptor agonist acetylcholine (ACh; 10 μM) in the presence of submaximal preconstriction (pretone) induced by the α_1_-adrenergic receptor agonist phenylephrine (10 μM).

### 4.10. Functional Studies on Isolated PAs

To examine endothelium-dependent and endothelium-independent vasorelaxation, CRCs to ACh (0.001–30 μM) and sodium nitroprusside (SNP, 0.0001–30 μM) were obtained, respectively, in PA rings preconstricted submaximally using the synthetic thromboxane A_2_ analog U-46619 (9,11-dideoxy-9α,11α-methanoepoxy prostaglandin F_2α_; 0.1–0.3 μM). Contractile function was assessed in isolated PAs exposed to increasing concentrations of 5-hydroxytryptamine (5-HT, 0.0001–30 μM). The vasodilatory effects of ACh and SNP were expressed as a percentage of relaxation relative to the isometric submaximal contraction induced by U-46619. Contractile responses to 5-HT were expressed as a percentage of the second exposure to KCl (50 mM). A comparable contractile arterial tone was observed in all examined PAs, as no differences were observed in response to KCl (50 mM) or U-46619 given before each experiment, similarly to Sadowska et al. [28]. In each individual ring, only one CRC was determined.

### 4.11. Histopathology

As a result of trimming the left lung transversely to the main airways, two lung fragments were obtained. The fragment of the heart’s RV was trimmed transversely to the heart muscle fibers, which led to obtaining two separate fragments—one was sectioned to provide a transverse view of cardiomyocytes, and the other one was a longitudinal section of cardiomyocytes. Both samples were processed in an automatic tissue processor through a series of alcohols and xylene to paraffin (Leica TP1020, Leica Biosystems Nussloch GmbH, Nussloch, Germany), according to standard histological paraffin embedding protocols. The samples were then embedded in paraffin blocks and sectioned to 3 μm using a rotating histological microtome. Routine hematoxylin-eosin (HE) staining was used for primary staining.

Histological slides were scanned with two microscope slide scanners (Ocus 20, Grundium, Tampere, Finland; Pannoramic 250 FLASH III, 3DHISTECH Kft., Budapest, Hungary). Quantitative morphometric measurements were performed using software for bioimaging analysis QuPath v0.5.1 [73]. Measurements were performed by pathologists—Doctors of Veterinary Medicine. In each HE lung histological slide, area of arteries’ tunica media was measured and presented as percentage of whole vessel area. The measurements included PAs < 250 μm in diameter. Exclusively, the vessels presented with transverse sections were measured.

In each HE heart histological slide, the diameter of an average 100 cardiomyocytes was measured. Cardiomyocytes were selected in both processed RV fragments so as to (i) be presented in the transverse section and (ii) be spread out evenly in both fragments concerning their placement within myocardium. The obtained results were presented in the form of mean cardiomyocyte diameter, according to adopted methodology [74].

### 4.12. Statistical Analysis

The individual rat was considered the experimental unit within the studies. Results are expressed as mean values ± standard error of the mean (SEM). Due to (1) high mortality of animals and (2) a few cases of failure in measurement of hemodynamic/ex vivo parameters, the number of results in each group is not uniform. The sample size was estimated on the basis of our previous experiments in this model, the available literature, and guidelines by Curtis et al. [75]. As not all outcomes were continuous numerical variables and within-Set mortality rate was difficult to predict, no a priori sample size calculation was performed. The CRCs were used to determine the potency (the pEC_50_: the negative logarithm of the concentration causing the half-maximum effect) and the maximum effect values (E_max_). All data were subjected to the Kolmogorov–Smirnov test to assess the distribution of values. If the data were normally distributed, the (parametric) one-way analysis of variance (ANOVA) with Bonferroni’s multiple comparison test for multiple groups or paired Student’s *t*-test for comparison within the group was carried out. Data subjected to ANOVA were followed by Bonferroni’s post hoc test only when the F value attained *p* < 0.05 and there was no significant inhomogeneity of variances. If the data were not normally distributed, the (non-parametric) Kruskal–Wallis test with Dunn’s multiple comparison test for multiple groups or paired Wilcoxon test for comparison within the group was carried out. Dunn’s post hoc test was only used when the Kruskal–Wallis test yielded a significant result (*p* < 0.05). The survival rate was calculated using the Kaplan–Meyer method, and the survival curves were compared with each other using the Gehan–Breslow–Wilcoxon test with Bonferroni’s post hoc analysis. Pearson’s correlation coefficients (r) were calculated to explore the correlation between the initial BW and PH development. The strength of the data correlation was categorized according to de Castro et al. [76], i.e., weak correlation (r = 0.20 to 0.39), moderate correlation (r = 0.40 to 0.69), and strong correlation (r = 0.70 to 0.89). Statistical analysis was performed using Graph Pad Prism 5 and 10 (GraphPad Software, La Jolla, CA, USA).

### 4.13. Drugs

Ambrisentan (PA-07-46395) and tadalafil (PA-03-0380-P) from POL-AURA, Zawroty, Poland; monocrotaline (C2401), (−)-isoprenaline (+)-bitartrate salt (I2760), acetylcholine chloride (A6625), (R)-(−)-phenylephrine hydrochloride (P6126), sodium nitroprusside dihydrate (567538), and 5-hydroxytryptamine creatinine sulfate complex (H7752) from Sigma-Aldrich, Burlington, MA, USA; U-46619 ((5Z)-7-[(1R,4S, 5S,6R)-6-[(1E,3S)-3-hydroxy-1-octenyl]-2-xabicyclo[2.2.1]hept5-yl]-5-heptenoic acid) (1932) from Tocris, Bristol, UK; isoflurane (5909991284336) from Vetpharma Animal Health, S.L., Barcelona, Spain; ketamine (5909997022796) from Biowet, Puławy, Poland; xylazine (5909997021911) from Vetoquinol Biowet, Gorzów Wielkopolski, Poland; and lidocaine (5909990937615) from EGIS Pharmaceuticals PLC, Budapest, Hungary.

Stock solutions of ISO were prepared using distilled water. Further dilutions were made with Tyrode’s solution. ACh, phenylephrine, and SNP were dissolved in deionized water and 5-HT in distilled water with a few drops of HCl. U-46619 was supplied in methyl acetate, which was evaporated under a stream of nitrogen, and ethanol was added as a solvent. Then, it was diluted to final concentrations with deionized water.

## 5. Conclusions

We demonstrated that both the severity of MCT-induced PH in male Wistar rats and the effectiveness of the reference combined therapy with AMB + TAD are strictly dependent on the initial BW of animals, even when relatively small differences exist. Older rats are less sensitive to PH development, and their response to therapy against PH is less pronounced. While PH development in younger rats is more severe, it is also associated with increased mortality. Furthermore, all the above findings are based on a single experiment performed under exactly the same conditions, which excludes other confounding factors identified so far. Unfortunately, our methodological study did not examine detailed mechanisms of the attained differences; thus, further research may be needed.

Considering our main insights, we propose that the initial BW should be mandatorily incorporated into the detailed standards and methodological rigor for preclinical and translational PH research, as outlined by Provencher et al. [19] (see Section 1). We do not postulate any particular body range as the best for preclinical studies, since it also depends on other factors described previously. Thus, it would be reasonable to perform compulsory preliminary experiments to determine the most optimal weight range for a particular rat strain, sex, experimental model, and/or other experiential expectations (e.g., early or late stage of PH) and to include the animals’ initial BW in the methods section along with age, which is often overlooked nowadays. We believe that our insights will contribute to a better reproducibility of preclinical experiments and facilitate their more effective translation into clinical trials. Additionally, it may help reduce the number of failed experiments due to an inefficiently planned methodology and thus reduce the number of animals used in research.

## Figures and Tables

**Table 1:** Influence of pulmonary hypertension (PH) and treatment with a combination of ambrisentan (AMB) and tadalafil (TAD) or their vehicle (veh) on physiological parameters in monocrotaline (MCT)-induced PH rats and their controls (CTR) across three weight Sets (Set I, Set II, and Set III, based on animal body weight on day 0—the PH induction).

		Set I 200–219 g			Set II 220–239 g			Set III 240–259 g		
		CTR + veh	MCT + veh	MCT + AMB + TAD	CTR + veh	MCT + veh	MCT + AMB + TAD	CTR + veh	MCT + veh	MCT + AMB + TAD
*n*		*4*	*3–9*	*5–8*	*9–10*	*5–9*	*6–10*	*6*	*7–8*	*7–8*
Body weight (BW) (g)	day 0	216 ± 2	213 ± 1	211 ± 2	228 ± 2	229 ± 2 ^###^	224 ± 1 ^###^	250 ± 3 ^### @^	252 ± 2 ^### @@@^	250 ± 1 ^### @@@^
	day 29	335 ± 9	284 ± 17 * ^$^	279 ± 6 ^$$$^	319 ± 5 ^$$$^	282 ± 10 ** ^$$^	313 ± 10 ^Δ $$$^	339 ± 3 ^$$$^	296 ± 6 *** ^$$$^	308 ± 10 ^$$^
Tibia length (TL) (mm)		37.3 ± 0.1	36.2 ± 0.4	36.2 ± 0.3	36.8 ± 0.2	36.8 ± 0.4	37.4 ± 0.2 ^##^	37.1 ± 0.1	36.7 ± 0.2	37.2 ± 0.2 ^#^
Heart rate (beats/min)	by echo (day 28)	439 ± 13	389 ± 24	398 ± 16	440 ± 6	377 ± 23 *	410 ± 16	446 ± 10	429 ± 10	410 ± 24
	by catheter	290 ± 17 °°°	317 ± 38	300 ± 24	279 ± 10 °°°	274 ± 20 °	282 ± 9 °°°	307 ± 18 °°°	271 ± 16 °°°	255 ± 26 °°°
dP/dt_max_ (mmHg/s)		1734 ± 107	3427 ± 50 **	2803 ± 275	1682 ± 51	3365 ± 340 ***	2244 ± 247 ^ΔΔ^	1490 ± 55	2733 ± 260 **	2183 ± 298
dP/dt_min_ (mmHg/s)		−1328 ± 117	−2520 ± 50 ***	−1805 ± 120 ^ΔΔ^	−1171 ± 43	−2547 ± 231 ***	−1561 ± 189 ^ΔΔΔ^	−1006 ± 56 ^#^	−2052 ± 134 ***	−1579 ± 201
Heart weight (mg)		1176 ± 44	1206 ± 80	1207 ± 65	1037 ± 41	1434 ± 42 ***	1378 ± 93	1067 ± 48	1170 ± 54	1253 ± 37
Heart weight/BW (mg/g)		3.5 ± 0.1	4.5 ± 0.4	4.3 ± 0.3	3.2 ± 0.1	5.1 ± 0.3 ***	4.5 ± 0.4	3.1 ± 0.1	4.0 ± 0.2	4.1 ± 0.2
Heart weight/TL (mg/mm)		32 ± 1	34 ± 2	33 ± 2	28 ± 1	39 ± 1 ***	37 ± 2	29 ± 1	32 ± 1	34 ± 1
RV weight (mg)		171 ± 14	324 ± 22	270 ± 30	151 ± 6	361 ± 13 ***	277 ± 28 ^ΔΔ^	159 ± 6	272 ± 27	287 ± 24
RV weight/BW (mg/g)		0.51 ± 0.03	1.05 ± 0.14 *	1.02 ± 0.12	0.47 ± 0.01	1.32 ± 0.08 ***	0.90 ± 0.10	0.47 ± 0.02	0.93 ± 0.10 ** ^@^	0.95 ± 0.11
RV weight/TL (mg/mm)		4.6 ± 0.4	8.1 ± 1.0 *	7.8 ± 0.8	4.1 ± 0.1	9.8 ± 0.4 ***	7.4 ± 0.7 ^ΔΔ^	4.3 ± 0.2	7.4 ± 0.7 **	7.7 ± 0.6
RA weight (mg)		54 ± 6	66 ± 17	47 ± 3	52 ± 5	87 ± 15	69 ± 11	44 ± 4	52 ± 6	57 ± 7
RA weight/BW (mg/g)		0.16 ± 0.02	0.23 ± 0.06	0.16 ± 0.02	0.16 ± 0.01	0.32 ± 0.07	0.22 ± 0.04	0.13 ± 0.01	0.18 ± 0.02	0.19 ± 0.03
RA weight/TL (mg/mm)		1.4 ± 0.2	1.7 ± 0.3	1.3 ± 0.1	1.4 ± 0.1	2.4 ± 0.4	1.8 ± 0.3	1.2 ± 0.1	1.4 ± 0.2	1.5 ± 0.2

AMB (10 mg/kg) and TAD (10 mg/kg) were administered by oral gavage once daily for 21 days, starting on day 8 from PH induction; veh groups received vehicle instead. Parameters were determined 24 h after the last dose, i.e., on day 29 (if not stated otherwise). Data are expressed as the means ± SEM; *n*—the number of rats per group. *^,∆,#,@,$,^° *p* < 0.05; **^,∆∆,##,$$^ *p* < 0.01; ***^,∆∆∆,###,@@@,$$$,^°°° *p* < 0.001—significant differences from * CTR + veh or ^∆^ MCT + veh within a given Set; from appropriate group in ^#^ Set I (200–219 g) and ^@^ Set II (220–239 g); from ^$^ day 0; and from the value from the ° echocardiographic measurement. Abbreviations: dP/dt_max_, dP/dt_min_, the rate of rise/decrease in right ventricular pressure; RA, right atrium; and RV, right ventricle.

**Table 2:** Summary of the significant changes in monocrotaline (MCT)-induced pulmonary hypertensive (PH) rats, and the effects of a combination of ambrisentan (AMB) and tadalafil (TAD) across three weight Sets (Set I, Set II, and Set III, based on the rats’ body weight on day 0—the PH induction).

			Influence of MCT in Comparison to Respective Control						Influence of AMB + TAD on the MCT-Induced PH					
			Set I 200–219 g		Set II 220–239 g		Set III 240–259 g		Set I 200–219 g		Set II 220–239 g		Set III 240–259 g	
BW gain			↓	40	↓	42	↓	51	↔		↑	68	↔	
Mortality [%]			67		22		13		38		38		10	
RVSP			↑	208	↑	219	↑	155	↓	36	↓	54	↔	
mPAP			↑	110	↑	76	↑	37	↓	23	↓	28	↔	
SpO_2_			↔		↓	13	↔		↔		↑	15	↔	
Heart hypertrophy	Fulton’s index		↑	92	↑	135	↑	77	↔		↓	26	↔	
	Heart weight		↔		↑	38	↔		↔		↔		↔	
	Heart weight/BW		↑	ns	↑	59	↑	ns	↔		↓	ns	↔	
	Heart weight/TL		↔		↑	39	↔		↔		↔		↔	
	RV weight		↑	ns	↑	139	↑	ns	↓	ns	↓	23	↔	
	RV weight/BW		↑	106	↑	181	↑	98	↔		↓	ns	↔	
	RV weight/TL		↑	76	↑	139	↑	72	↔		↓	24	↔	
	RV wall thickness in diastole		↑	78	↑	124	↑	53	↔		↓	38	↔	
	RV wall thickness in systole		↑	55	↑	85	↑	54	↔		↓	34	↔	
	RV cardiomyocytes width		↑	23	↑	12	↑	21	↓	16	↓	10	↓	7
	RA weight		↔		↑	ns	↔		↓	ns	↓	ns	↔	
	RA weight/BW		↑	ns	↑	ns	↑	ns	↓	ns	↓	ns	↔	
	RA weight/TL		↔		↑	ns	↔		↓	ns	↓	ns	↔	
Heart function	rise in dP/dt_max_		↑	97	↑	100	↑	83	↓	ns	↓	33	↓	ns
	decrease in dP/dt_min_		↑	90	↑	118	↑	104	↓	28	↓	39	↓	ns
	HR (catheter)		↔		↔		↓	ns	↔		↔		↔	
	HR (echo)		↓	ns	↓	14	↓	ns	↔		↔		↔	
	RV end-diastolic area		↑	ns	↑	119	↑	92	↓	ns	↓	34	↔	
	RV end-systolic area		↑	ns	↑	155	↑	144	↔		↓	34	↔	
	RV fractional area change		↓	ns	↓	ns	↓	ns	↔		↔		↔	
	RV internal diameter in diastole		↑	ns	↑	15	↑	16	↔		↔		↔	
	RV internal diameter in systole		↑	54	↑	47	↑	48	↔		↔		↔	
	RV fractional shortening		↓	54	↓	54	↓	54	↔		↔		↔	
	RV end-diastolic volume		↑	ns	↑	40	↑	53	↓	ns	↔		↔	
	RV end-systolic volume		↑	268	↑	182	↑	204	↓	ns	↓	ns	↔	
	RV stroke volume		↓	22	↓	30	↓	19	↔		↔		↔	
	RV ejection fraction		↓	47	↓	42	↓	46	↔		↔		↔	
	RV cardiac output		↓	ns	↓	38	↓	ns	↔		↔		↔	
	TAPSE		↓	30	↓	33	↓	25	↔		↔		↔	
	RV papillary muscles baseline developed tension		↔		↑	167	↔		↔		↓	64	↔	
	RV papillary muscles response to ISO	pEC_50_ (% basal and developed tension)	↔		↔		↔		↔		↔		↔	
		E_max_ (% basal)	↓	ns	↓	50	↓	ns	↑	ns	↑	123	↑	87
		E_max_ (developed tension)	↔		↔		↔		↑	ns	↔		↑	173
Lung and PA hypertrophy and function	Lung hypertrophy		↑	63	↑	62	↑	48	↔		↓	ns	↔	
	Muscularization of PA		↑	16	↑	17	↑	13	↔		↓	14	↔	
	PA acceleration time (AT)		↓	54	↓	44	↓	24	↑	52	↑	51	↔	
	PA ejection time (ET)		↓	18	↓	22	↔		↔		↑	ns	↔	
	PA AT/ET		↓	44	↓	32	↓	ns	↑	41	↑	ns	↔	
	PA velocity time integral		↓	49	↓	47	↓	36	↔		↑	43	↔	
	PA internal diameter		↔		↑	23	↔		↔		↓	15	↔	
	PA response to ACh	pEC_50_	↔		↓	11	↓	14	↓	15	↑	27	↑	7
		E_max_	↔		↔		↔		↔		↑	ns	↑	45
	PA response to SNP	pEC_50_	↓	6	↓	14	↔		↔		↑	15	↑	7
		E_max_	↑	49	↑	ns	↔		↓	ns	↔		↑	73
	PA response to 5-HT	pEC_50_	↔		↔		↔		↑	11	↔		↑	11
		E_max_	↑	67	↑	38	↑	ns	↑	ns	↔		↑	ns

↑, increase; ↓, decrease; ↔, no effect; and ns, non-significant tendency. The numbers next to the arrows represent the percentage change in the parameter (except for mortality, presented as absolute values). The cell colors indicate the intensity of the observed differences: unfavorable changes are shown in shades of blue, while beneficial changes are represented in shades of purple. The percentage changes are derived from the data presented in Figure 1, Figure 2, Figure 3, Figure 4, Figure 5, Figure 6 and Figure 7 and Table 1, Tables S2 and S3. Abbreviations: ACh, acetylcholine; BW, body weight; dP/dt_max_, dP/dt_min_, rate of rise/decrease in right ventricular pressure; E_max_, maximum effect; HR, heart rate; 5-HT, 5-hydroxytryptamine; ISO, isoprenaline; mPAP, mean pulmonary artery pressure; PA, pulmonary artery; pEC_50_, the negative logarithm of the concentration causing the half-maximum effect; RA, right atrium; RV, right ventricle; RVSP, RV systolic pressure; SNP, sodium nitroprusside; SpO_2_, blood oxygen saturation; TAPSE, tricuspid annular plane systolic excursion; and TL, tibia length.

## Data Availability

Data generated or analyzed during this study are available from the corresponding author upon reasonable request.

## Supplementary Materials

The following supporting information can be downloaded at: https://www.mdpi.com/article/10.3390/ijms26188916/s1.

## Abbreviations

The following abbreviations are used in this manuscript:

5-HT	5-Hydroxytryptamine
ACh	Acetylcholine
AMB	Ambrisentan
ANOVA	Analysis of variance
BW	Body weight
CO	Cardiac output
CRC	Concentration-response curve
CTR	Control
dP/dt_max_	The rate of rise in right ventricular pressure
dP/dt_min_	The rate of decrease in right ventricular pressure
EDA	End-diastolic area
EDV	End-diastolic volume
EF	Ejection fraction
E_max_	Maximum effect
ESA	End-systolic area
ESV	End-systolic volume
ET	Ejection time
ET-1	Endothelin 1
FAC	Fractional area change
FS	Fractional shortening
HE	Hematoxylin and eosin
HR	Heart rate
IDD	Internal diameter in diastole
IDS	Internal diameter in systole
ISO	Isoprenaline
LA	Left atrium
LV	Left ventricle
LV + S	Left ventricle with septum
LVWTd	Left ventricular wall thickness in diastole
LVWTs	Left ventricular wall thickness in systole
MCT	Monocrotaline
mPAP	Mean aulmonary artery pressure
PA	Pulmonary artery
PAAT	Pulmonary artery acceleration time
PAH	Pulmonary arterial hypertension
pEC_50_	Negative logarithm of the concentration causing the half-maximum effect
PH	Pulmonary hypertension
PDE-5	Phosphodiesterase 5
RA	Right atrium
RV	Right ventricle
RVOT	Right ventricular outflow tract
RVSP	Right ventricular systolic pressure
RVWTd	Right ventricular wall thickness in diastole
RVWTs	Right ventricular wall thickness in systole
SEM	Standard error of the mean
SNP	Sodium nitroprusside
SpO_2_	Blood oxygen saturation
SV	Stroke volume
TAD	Tadalafil
TAPSE	Tricuspid annular plane systolic excursion
TL	Tibia length
veh	Vehicle
VTI	Velocity time integral
