# Supplementary material for: Initial Body Weight as an Important Factor for Improving the Reliability and Translational Relevance of the Preclinical Monocrotaline-Induced Rat Pulmonary Hypertension Model
Source: Int J Mol Sci. 2025 Sep 12;26(18):8916. doi: 10.3390/ijms26188916 (PMC12469526; doi:10.3390/ijms26188916)
Supplement: Supplementary file 1 [file ijms-26-08916-s001.zip › Figure S1.pdf]

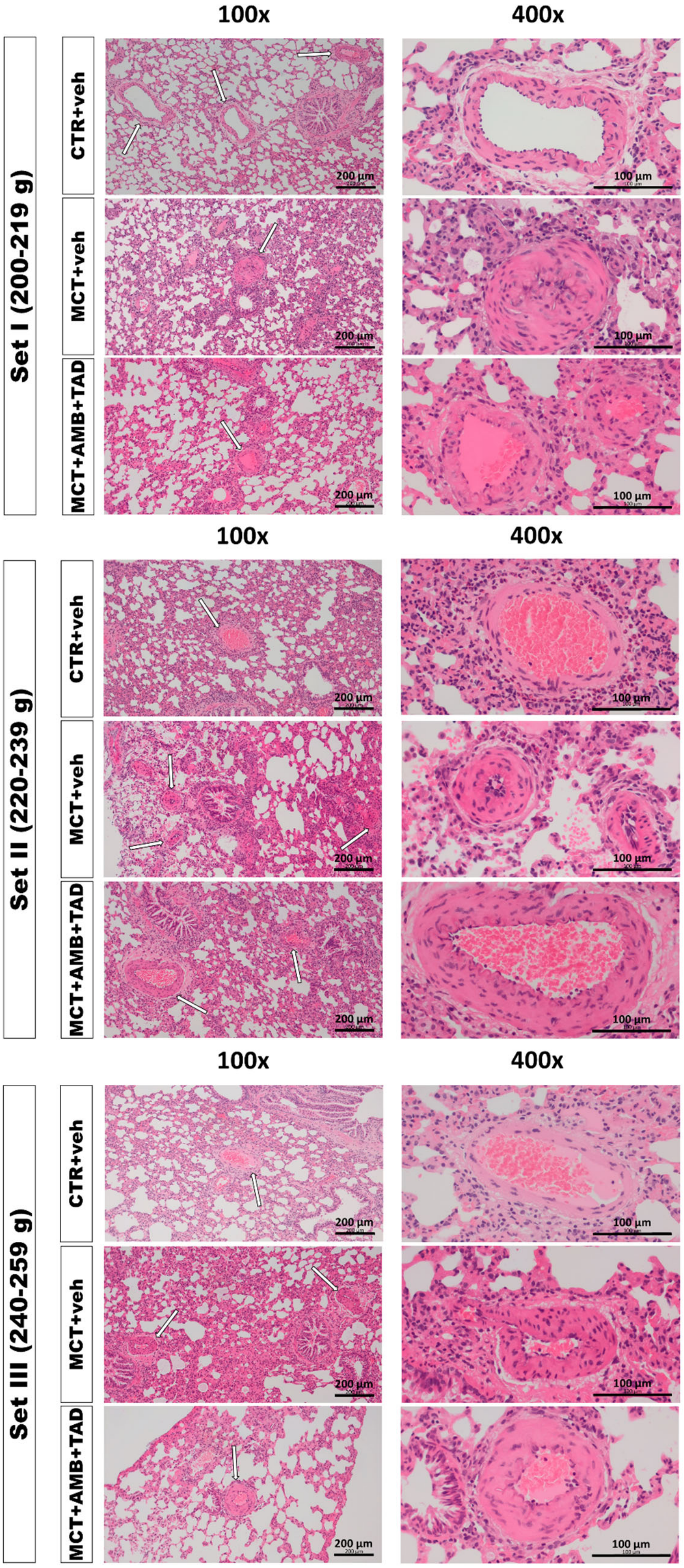

Figure S1. Influence of pulmonary hypertension (PH) and treatment with a combination of ambrisentan (AMB) and tadalafil (TAD) or their vehicle (veh) on vascular remodeling in monocrotaline (MCT)-induced PH rats and their controls (CTR) across three weight sets (Set I, Set II, and Set III, based on animal body weight on day 0 – the PH induction). The figure shows representative images of left lungs stained with hematoxylin and eosin (100x and 400x magnification). The percentage muscularization of the pulmonary arteries (PA) calculated using histological images can be found in Figure 6. AMB (10 mg/kg) and TAD (10 mg/kg) were administered by oral gavage once daily for 21 days, starting on day 8 from PH induction; veh groups received vehicle instead.
