# Supplementary material for: Initial Body Weight as an Important Factor for Improving the Reliability and Translational Relevance of the Preclinical Monocrotaline-Induced Rat Pulmonary Hypertension Model
Source: Int J Mol Sci. 2025 Sep 12;26(18):8916. doi: 10.3390/ijms26188916 (PMC12469526; doi:10.3390/ijms26188916)
Supplement: Supplementary file 1 [file ijms-26-08916-s001.zip › Tables S1-S5.pdf]

Table S1. The correlations between initial body weight (BW) of monocrotaline (MCT)-induced pulmonary hypertensive (PH) rats and the four parameters used to assess disease severity.

	95% CI	p value	r	Correlation strength
**MCT+veh**				
Initial BW × mPAP	-0.9040 to -0.4727	0.0001	-0.7631	strong
Initial BW × RVSP	-0.7808 to 0.08409	0.0944	-0.4474	no correlation
Initial BW × FI	-0.5658 to 0.3547	0.5950	-0.1344	no correlation
Initial BW × SpO_2	-0.5561 to 0.3670	0.6339	-0.1205	no correlation
**MCT+AMB+TAD**				
Initial BW × mPAP	-0.4654 to 0.4190	0.9040	-0.02882	no correlation
Initial BW × RVSP	-0.5344 to 0.3934	0.7226	-0.08995	no correlation
Initial BW × FI	-0.5014 to 0.4043	0.8038	-0.06110	no correlation
Initial BW × SpO_2	-0.8212 to -0.2068	0.0057	-0.5948	moderate

The strength of the data correlation was categorized based on the absolute value of r, with the following classifications: weak correlation (r = 0.20 to 0.39), moderate correlation (r = 0.40 to 0.69), and strong correlation (r = 0.70 to 0.89) [76].

Abbreviations: AMB, ambrisentan; CI, confidence interval; FI, Fulton index; mPAP, mean pulmonary artery pressure; RVSP, right ventricular systolic pressure; SpO_2, blood oxygen saturation; TAD, tadalafil; veh, vehicle.

Table S2. Influence of pulmonary hypertension (PH) and treatment with a combination of ambrisentan (AMB) and tadalafil (TAD) or their vehicle (veh) on the isoprenaline (0.0001 – 10 µM)-induced positive inotropic effects in right ventricular papillary muscles isolated form monocrotaline (MCT)-induced PH rats and their controls (CTR) across three weight sets (Set I, Set II, and Set III, based on animal body weight on day 0 – the PH induction).

	**Set I (200-219 g)**			**Set II (220-239 g)**			**Set III (240-259 g)**		
	**CTR +veh**	**MCT +veh**	**MCT +AMB +TAD**	**CTR +veh**	**MCT +veh**	**MCT +AMB +TAD**	**CTR +veh**	**MCT +veh**	**MCT +AMB +TAD**
n	*4*	*3*	*3*	*7*	*4*	*4*	*6*	*5*	*4*
**force (% of basal)**									
pEC_{50}	7.82 ± 0.54	7.75 ± 0.34	7.58 ± 0.37	7.61 ± 0.19	7.79 ± 0.22	7.39 ± 0.21	7.63 ± 0.28	7.15 ± 0.23	7.55 ± 0.19
E_{max} [%]	56.17 ± 18.21	36.85 ± 1.58	66.87 ± 16.11	86.79 ± 9.86	43.21 ± 7.81**	96.36 ± 24.35 $^{\Delta}$	85.41 ± 17.80	52.84 ± 6.26	98.97 ± 13.49 $^{\Delta}$
**delta (mN/cm²)**									
pEC_{50}	7.62 ± 0.31	7.99 ± 0.32	7.51 ± 0.44	7.49 ± 0.29	7.61 ± 0.26	7.43 ± 0.38	7.35 ± 0.37	7.07 ± 0.41	7.56 ± 0.24
E_{max} [%]	7.19 ± 1.40	7.45 ± 2.59	16.24 ± 6.13	18.04 ± 4.84	21.31 ± 6.50	19.44 ± 7.66	12.09 ± 4.34	10.93 ± 4.64	29.88 ± 6.81 $^{\Delta}$

Values are based on the concentration-response curves shown in Fig. 4. AMB (10 mg/kg) and TAD (10 mg/kg) were administered by oral gavage once daily for 21 days, starting on day 8 from PH induction; veh groups received vehicle instead. Data are expressed as the means ± SEM; *n* = 3–7 rats per group; $^{\Delta}p < 0.05$; $^{**}p < 0.01$ – significant differences from *CTR + veh or $^{\Delta}$MCT + veh within a given Set.

Abbreviations: E_{max}, the maximum effect; pEC_{50}, the negative logarithm of the concentration causing the half-maximum effect.

Table S3. Influence of pulmonary hypertension (PH) and treatment with a combination of ambrisentan (AMB) and tadalafil (TAD) or their vehicle (veh) on responses to acetylcholine (ACh), sodium nitroprusside (SNP) and 5-hydroxytryptamine (5-HT) in pulmonary arteries isolated form monocrotaline (MCT)-induced PH rats and their controls (CTR) across three weight sets (Set I, Set II, and Set III, based on animal body weight on day 0 – the PH induction).

	**Set I (200-219 g)**			**Set II (220-239 g)**			**Set III (240-259 g)**		
	**CTR +veh**	**MCT +veh**	**MCT +AMB +TAD**	**CTR +veh**	**MCT +veh**	**MCT +AMB +TAD**	**CTR +veh**	**MCT +veh**	**MCT +AMB +TAD**
**ACh**									
pEC_{50}	6.2 ± 0.1	6.0 ± 0.1	5.1 ± 0.1 $^{\Delta\Delta\Delta}$	6.3 ± 0.1	5.6 ± 0.1 $^{***,\#}$	7.1 ± 0.1 $^{\Delta\Delta\Delta,\ \#\#\#}$	6.3 ± 0.1	5.4 ± 0.1 $^{***,\#\#}$	5.8 ± 0.1 $^{\Delta\Delta,\ \#\#\#,\ @@@}$
E_{max} [%]	74.5 ± 4.3	68.3 ± 9.6	68.4 ± 9.6	60.7 ± 6.5	62.8 ± 5.7	78.2 ± 7.0	64.1 ± 4.1	55.2 ± 6.1	79.8 ± 4.1 $^{\Delta\Delta}$
n	*5*	*6*	*5*	*5*	*8*	*3*	*5*	*6*	*6*
**SNP**									
pEC_{50}	6.9 ± 0.1	6.5 ± 0.1 *	6.6 ± 0.1	7.0 ± 0.1	6.0 ± 0.1 $^{***,\#\#}$	6.9 ± 0.1 $^{\Delta\Delta\Delta}$	6.7 ± 0.1	6.7 ± 0.1 $^{@@@}$	7.2 ± 0.1 $^{\Delta}$
E_{max} [%]	67.2 ± 8.4	100.1 ± 4.8 ***	83.2 ± 7.6	83.1 ± 2.4	98.8 ± 13.6	102.0 ± 13.6	58.7 ± 3.5 $^{@}$	58.7 ± 3.5 $^{\#\#\#,@}$	101.3 ± 4.6 $^{\Delta\Delta\Delta}$
n	*6*	*6*	*3*	*5*	*5*	*3*	*5*	*5*	*6*
**5-HT**									
pEC_{50}	5.5 ± 0.1	5.6 ± 0.1	6.2 ± 0.1 $^{\Delta\Delta}$	5.4 ± 0.1	5.6 ± 0.1	5.6 ± 0.1 $^{\#\#}$	5.5 ± 0.1	5.4 ± 0.1	6.0 ± 0.1 $^{\Delta\Delta,@}$
E_{max} [%]	54.1 ± 7.1	90.5 ± 1.7 ***	100.9 ± 3.7	67.0 ± 4.2	92.2 ± 5.3 **	84.7 ± 1.7 $^{\#}$	62.8 ± 8.6	78.3 ± 4.7	93.5 ± 2.5
n	*5*	*5*	*5*	*6*	*5*	*5*	*5*	*5*	*6*

Values are based on the concentration-response curves shown in Fig. 7. AMB (10 mg/kg) and TAD (10 mg/kg) were administered by oral gavage once daily for 21 days, starting on day 8 from PH induction; veh groups received vehicle instead. Data are expressed as the means ± SEM; *n* = 3–8 rats per group; $^{*,\Delta,\#,@}p < 0.05$; $^{**,\Delta\Delta,\#\#}p < 0.01$; $^{***,\Delta\Delta\Delta,\#\#\#,@@@}p < 0.001$ – significant differences from *CTR + veh or $^{\Delta}$MCT + veh within a given Set; and from appropriate group in $^{\#}$ Set I (200-219 g) and $^{@}$ Set II (220-239 g).

Abbreviations: E_{max}, the maximum effect; pEC_{50}, the negative logarithm of the concentration causing the half-maximum effect.

Table S4. Influence of pulmonary hypertension (PH) and treatment with a combination of ambrisentan (AMB) and tadalafil (TAD) on left ventricular (LV) parameters measured by echocardiography in three weight sets of monocrotaline (MCT)-induced PH rats and their controls (CTR).

		**Set I 200-219 g**			**Set II 220-239 g**			**Set III 240-259 g**		
		**CTR +veh**	**MCT +veh**	**MCT +AMB +TAD**	**CTR +veh**	**MCT +veh**	**MCT +AMB +TAD**	**CTR +veh**	**MCT +veh**	**MCT +AMB +TAD**
	n	*4*	*3-9*	*5-8*	*9-10*	*5-9*	*6-10*	*6*	*7-8*	*7-8*
	day									
LV wall thickness in diastole (mm)	7	1.54 ± 0.09	1.66 ± 0.13	1.75 ± 0.08	1.64 ± 0.05	1.61 ± 0.09	1.71 ± 0.05	1.71 ± 0.12	1.64 ± 0.07	1.53 ± 0.11
	28	1.75 ± 0.09	2.09 ± 0.64	1.62 ± 0.08	1.81 ± 0.11	1.80 ± 0.30	1.78 ± 0.09	1.83 ± 0.08	1.74 ± 0.06	1.87 ± 0.31
LV wall thickness in systole (mm)	7	3.27 ± 0.14	3.18 ± 0.21	3.32 ± 0.16	3.32 ± 0.07	3.17 ± 0.19	3.33 ± 0.05	3.45 ± 0.05	3.26 ± 0.15	3.30 ± 0.20
	28	3.52 ± 0.13	3.69 ± 0.34	3.41 ± 0.27	3.56 ± 0.13	3.51 ± 0.43	3.66 ± 0.18	3.45 ± 0.13	3.75 ± 0.17	3.41 ± 0.36
LV internal diameter in end diastole (mm)	7	5.12 ± 0.27	5.06 ± 0.14	5.34 ± 0.07	5.62 ± 0.08	5.49 ± 0.09	5.11 ± 0.13	5.40 ± 0.12	5.59 ± 0.06	5.53 ± 0.08
	28	5.44 ± 0.09	5.19 ± 0.14	5.46 ± 0.08	5.60 ± 0.06	4.99 ± 0.30	5.27 ± 0.13	5.63 ± 0.04	5.46 ± 0.12	5.37 ± 0.15
LV internal diameter in end systole (mm)	7	3.45 ± 0.17	3.27 ± 0.10	3.56 ± 0.07	3.74 ± 0.10	3.66 ± 0.09	3.29 ± 0.09	3.80 ± 0.14	3.72 ± 0.12	3.79 ± 0.07
	28	3.55 ± 0.01	3.90 ± 0.10 $	3.96 ± 0.15	3.73 ± 0.10	3.47 ± 0.27	3.58 ± 0.08	3.87 ± 0.12	4.20 ± 0.10	4.02 ± 0.12
LV end-diastolic volume (mm^3)	7	331 ± 47	316± 25	367 ± 13	423 ± 18	396 ± 18	328 ± 24	377 ± 23	416 ± 12	404 ± 16
	28	385 ± 18	339 ± 27	388 ± 15	414 ± 12	319 ± 48	356 ± 25	423 ± 7	391 ± 23	375 ± 26
LV end-systolic volume (mm^3)	7	107 ± 15	91 ± 7	116 ± 7	135 ± 11	127 ± 9	94 ± 7	141 ± 16	132 ± 11	138 ± 7 @
	28	114 ± 1	151 ± 11 $$	159 ± 18	134 ± 11	118 ± 23	118 ± 8	148 ± 13	181 ± 13 $	165 ± 14
LV stroke volume (mm^3)	7	223 ± 32	225 ± 18	251 ± 14	289 ± 10	265 ± 12	234 ± 17	236 ± 21	284 ± 5	266 ± 15
	28	271 ± 19	188 ± 26 *	229 ± 13	282 ± 8	177 ± 34 ** $	238 ± 19	276 ± 11	205 ± 13 ** $$	210 ± 20
LV ejection fraction (%)	7	67.5 ± 0.2	71.1 ± 1.2	68.1 ± 2.1	68.5 ± 1.6	68.1 ± 1.3	71.4 ± 0.7	62.5 ± 3.6	68.5 ± 2.0	65.7 ± 1.8
	28	70.1 ± 1.6	54.7 ± 4.0 * $	59.4 ± 3.4 $	68.0 ± 1.8	60.9 ± 4.6	66.7 ± 1.0	65.2 ± 2.7	52.3 ± 1.22 *** $$	55.6 ± 2.9
LV fractional shortening (%)	7	32.7 ± 0.2	35.3 ± 0.9	33.4 ± 1.5	33.6 ± 1.1	33.2 ± 1.0	35.5 ± 0.5	29.5 ± 2.5	33.6 ± 1.4	31.5 ± 1.3
	28	34.7 ± 1.2	24.7 ± 2.4 * $	27.6 ± 2.2 $	33.4 ± 1.3	34.4 ± 3.8	32.1 ± 0.8	31.3 ± 1.9	20.4 ± 2.6 ** $	25.2 ± 1.7
LV end-diastolic area (mm^2)	7	19.7 ± 2.4	20.7 ± 0.8	20.6 ± 0.8	21.8 ± 0.9	22.4 ± 2.3	17.8 ± 1.6	24.1 ± 1.0	22.7 ± 0.4	21.7 ± 1.3
	28	22.8 ± 0.7	22.3 ± 2.0	23.7 ± 0.9	24.9 ± 1.5	19.9 ± 3.0	20.7 ± 1.6	24.7 ± 0.6	21.7 ± 1.3	22.8 ± 1.9
LV end-systolic area (mm^2)	7	11.9 ± 1.5	11.6 ± 0.6	12.2 ± 0.8	11.1 ± 0.5	12.3 ± 1.6	9.2 ± 0.8	13.0 ± 0.3	13.4 ± 1.1	11.9 ± 0.5
	28	13.3 ± 0.6	14.4 ± 1.3	14.3 ± 1.0	12.0 ± 0.9	10.5 ± 1.6	11.8 ± 0.9	14.3 ± 0.7	13.9 ± 0.9	14.0 ± 0.8
LV fractional area change (%)	7	39.8 ± 2.7	43.9 ± 2.5	41.1 ± 2.2	49.5 ± 1.6	45.6 ± 2.4	48.9 ± 1.7	45.8 ± 2.2	41.1 ± 4.4	44.8 ± 1.20
	28	41.4 ± 3.3	36.2 ± 1.0	39.1 ± 4.9	49.8 ± 3.8	40.2 ± 2.8	42.4 ± 3.1	42.1 ± 2.6	37.2 ± 1.3	37.1 ± 4.1
LV cardiac output (ml/min)	7	102 ± 15	98 ± 10	108 ± 6	128 ± 6	112 ± 6	103 ± 8	109 ± 11	124 ± 4	119 ± 8
	28	119 ± 9	74 ± 12 *	92 ± 8	124 ± 5	67 ± 14 *** $$	97 ± 8	123 ± 6	90 ± 6 ** $	87 ± 11 $

AMB (10 mg/kg) and TAD (10 mg/kg) were administered by oral gavage once daily for 21 days, starting on day 8 from PH induction; veh groups received vehicle instead. Parameters were determined on days 7 and 28 from PH induction. Data are expressed as the means ± SEM, *n* – the number of rats per group; *,@,$p < 0.05; **,$$p < 0.01; ***p < 0.001 – significant differences from *CTR + veh within a given Set; from appropriate group in # Set I (200-219 g) and @ Set II (220-239 g); and from $ day 7.

Table S5. Influence of pulmonary hypertension (PH) and treatment with a combination of ambrisentan (AMB) and tadalafil (TAD) on selected physiological parameters in three weight sets of monocrotaline (MCT)-induced PH rats and their controls (CTR).

	**Set I 200-219 g**			**Set II 220-239 g**			**Set III 240-259 g**		
	**CTR +veh**	**MCT +veh**	**MCT +AMB +TAD**	**CTR +veh**	**MCT +veh**	**MCT +AMB +TAD**	**CTR +veh**	**MCT +veh**	**MCT +AMB +TAD**
n	*4*	*3-9*	*5-8*	*9-10*	*5-9*	*6-10*	*6*	*7-8*	*7-8*
LV+S weight (mg)	672 ± 24	587 ± 42	589 ± 15	598 ± 21	608 ± 11	633 ± 24	611 ± 14	583 ± 12	632 ± 17
LV+S weight/BW (mg/g)	2.01 ± 0.07	2.14 ± 0.05	2.06 ± 0.03	1.87 ± 0.05	2.21 ± 0.07 **	2.03 ± 0.09	1.80 ± 0.04	1.98 ± 0.07	2.06 ± 0.06
LV+S weight/TL (mg/mm)	18 ± 1	17 ± 1	16 ± 0	16 ± 1	17 ± 0	17 ± 1	16 ± 0	16 ± 0	17 ± 0
LA weight (mg)	34 ± 1	16 ± 2	23 ± 1	24 ± 2	21 ± 2	27 ± 2	27 ± 2	21 ± 2	25 ± 5
LA weight/BW (mg/g)	0.10 ± 0.01	0.06 ± 0.00	0.08 ± 0.00	0.07 ± 0.01	0.08 ± 0.01	0.09 ± 0.01	0.08 ± 0.00	0.07 ± 0.00	0.08 ± 0.01
LA weight/TL (mg/mm)	0.9 ± 0.0	0.5 ± 0.1	0.7 ± 0.0	0.6 ± 0.1	0.6 ± 0.1	0.7 ± 0.1	0.7 ± 0.0	0.6 ± 0.0	0.7 ± 0.1
Kidney weight (mg)	1273 ± 26	993 ± 57	1036 ± 40	1213 ± 35	1064 ± 48	1198 ± 53	1204 ± 49	1026 ± 36	1142 ± 85
Kidney weight/BW (mg/g)	3.8 ± 0.2	3.7 ± 0.1	3.6 ± 0.1	3.8 ± 0.1	3.8 ± 0.1	3.8 ± 0.1	3.6 ± 0.1	3.5 ± 0.1	3.7 ± 0.2
Kidney weight/TL (mg/mm)	34 ± 1	28 ± 1	29 ± 1	33 ± 1	29 ± 1	32 ± 1	32 ± 1	28 ± 1	31 ± 2
Glucose (mg/dL)	141 ± 5	109 ± 20	139 ± 9	131 ± 5	110 ± 13	127 ± 12	137 ± 7	118 ± 5	146 ± 14
Triglycerides (mg/dL)	156 ± 29	145 ± 13	121 ± 4	164 ± 7	138 ± 18	136 ± 16	164 ± 25	126 ± 14	157 ± 20
Cholesterol (mg/dL)	159 ± 2	160 ± 3	161 ± 4	157 ± 1	159 ± 3	158 ± 2	157 ± 1	158 ± 2	156 ± 2
Lactate (mmol/L)	3.8 ± 0.5	5.0 ± 1.9	4.9 ± 0.7	4.0 ± 0.7	5.9 ± 1.2	5.5 ± 1.6	3.9 ± 0.4	4.7 ± 0.8	5.8 ± 0.8

AMB (10 mg/kg) and TAD (10 mg/kg) were administered by oral gavage once daily for 21 days, starting on day 8 from PH induction; veh groups received vehicle instead. Parameters were determined 24 h after the last dose, i.e. on day 29. Data are expressed as the means ± SEM, *n* – the number of rats per group; **p < 0.01 – significant difference from CTR+veh within a given Set.

Abbreviations: BW, body weight; LA, left atrium; LV+S, left ventricle with septum; TL, tibia length.
